# mdRNA-Seq analysis of marine microbial communities from the northern Red Sea

**DOI:** 10.1038/srep35470

**Published:** 2016-10-19

**Authors:** Shengwei Hou, Ulrike Pfreundt, Dan Miller, Ilana Berman-Frank, Wolfgang R. Hess

**Affiliations:** 1 Genetics and Experimental Bioinformatics, Faculty of Biology, University of Freiburg, Schänzlestr. 1, 79104 Freiburg, Germany; 2 Mina and Everard Goodman Faculty of Life Sciences, Bar-Ilan University, Ramat Gan 52900, Israel

## Abstract

Metatranscriptomic differential RNA-Seq (mdRNA-Seq) identifies the suite of active transcriptional start sites at single-nucleotide resolution through enrichment of primary transcript 5′ ends. Here we analyzed the microbial community at 45 m depth at Station A in the northern Gulf of Aqaba, Red Sea, during 500 m deep mixing in February 2012 using mdRNA-Seq and a parallel classical RNA-Seq approach. We identified promoters active *in situ* for five different pico-planktonic genera (the SAR11 clade of *Alphaproteobacteria*, *Synechococcus* of Cyanobacteria, *Euryarchaeota*, *Thaumarchaeota*, and *Micromonas* as an example for picoeukaryotic algae), showing the applicability of this approach to highly diverse microbial communities. 16S rDNA quantification revealed that 24% of the analyzed community were group II marine *Euryarchaeota* in which we identified a highly abundant non-coding RNA, Tan1, and detected very high expression of genes encoding intrinsically disordered proteins, as well as enzymes for the synthesis of specific B vitamins, extracellular peptidases, carbohydrate-active enzymes, and transport systems. These results highlight previously unknown functions of *Euryarchaeota* with community-wide relevance. The complementation of metatranscriptomic studies with mdRNA-Seq provides substantial additional information regarding transcriptional start sites, promoter activities, and the identification of non-coding RNAs.

Metatranscriptomics is widely used to study microbial community functions and especially marine microbial communities^1^,^2^,^3^,^4^,^5^,^6^. Differential RNA-Seq (dRNA-Seq) is a technique that enriches the primary transcript 5′ ends based on the presence of the triphosphate remaining from the initiation of transcription in bacteria or based on the eukaryotic 5′ cap, which can be converted to ligatable ends using de-capping enzymes^7^. The key advantage of this methodology is that it generates a genome-wide map of all transcriptional start sites (TSSs) that were active at the time of sampling at single nucleotide-resolution, and yields specific information about non-coding transcripts. Accordingly, dRNA-Seq has been widely used to infer the primary transcriptomes of bacteria in culture^7^,^8^,^9^,^10^. Recently, a generic “dual RNA-seq” approach has been introduced in which this methodology was applied to a bacterial pathogen and its eukaryotic host^11^. However, this methodology has not been applied on environmental microbial communities.

The Red Sea is characterized by high temperatures and evaporation rates, extremely low precipitation and riverine inputs, high salinities, and is thermally stratified from April-November^12^. The Gulf of Aqaba/Eilat (hereafter the Gulf) is connected to the Red Sea only by the Straits of Tiran^13^. As a consequence, warm and saline surface waters enter the Gulf while minimal temperatures below the thermocline remain at 20.6 °C^14^. During the winter months, surface water temperatures decline, destabilizing the thermocline, and deepening the mixed layer which can typically reach 300 to 800 m^15^. Many features of the Red Sea^16^,^17^,^18^,^19^, as well as the Gulf 20,21,22,23,24,25,26,27,28,29, have been investigated including taxonomic, physiological, and genetic aspects. Metagenomic and metatranscriptomic approaches were used to characterize the different microbial rhodopsins in the Red Sea^30^ and to target the most strongly expressed functions of the microbiome associated with the sponge *Stylissa carteri*^31^. However, metatranscriptomic profiling has not been used extensively thus far to characterize the Red Sea planktonic microbial community.

Here, we have applied metatranscriptomic dRNA-Seq (mdRNA-Seq) to the analysis of a sample obtained from a depth of 45 m from Station A at the northern tip of the Gulf (29°28′N 34°55′E, bottom depth ~700 m) during winter deep mixing conditions^32^,^33^. We chose these conditions to address a highly diverse marine microbial population with mdRNA-Seq, a new technique, and performed in parallel a classical metatranscriptomic (RNA-Seq) approach to compare the output from the two methods. We addressed the composition of the microbial community by 16S analysis, and studied its activity by applying mdRNA-Seq and a parallel classical (RNA-Seq) approach to evaluate their analytical power. We summarize the main principles and specificities of the two methods in Fig. 1.

Our specific objectives in this study were:

- To test the feasibility of mdRNA-Seq for the characterization of complex marine communities consisting of microorganisms from the three domains of life;
- To present some specific examples of knowledge generated by using mdRNA-Seq, and
- To place these results into a broader context of microbial activities within the marine realm.

## Results and Discussion

### Environmental conditions and composition of the microbial community at 45 m

On the day of sampling, water temperature at 45 m depth was 21.3 °C, salinity 40.7 PSU. Water contained 187–188 μM of dissolved oxygen, 1.85 μM inorganic nitrogen (NO_3_ + NO_2_), 0.1 μM inorganic phosphorus and 0.11 μgL^−1^ of chlorophyll *a*. Details of how these values changed along the water column were published earlier along with the dataset^32^.

We elucidated the composition of the microbial community by 16S rDNA quantification. *Proteobacteria* was the most abundant phylum, comprising 42.08% of all reads, followed by *Euryarchaeota* and *Cyanobacteria/Chloroplast*, which accounted for 25.06% and 10.08% of total reads, respectively (Fig. 2a). The taxa were further specified to family level, depending on the available OTU classification (Fig. 2b). This analysis demonstrated that reads assigned to *Proteobacteria* belonged to 12 different families, with relative abundances increasing from 0.72% for uncultured *Myxococcales* to 6.25% for *Rhodospirillaceae* (Fig. 2b). Within the *SAR11_clade* (altogether 7.13% of all reads), *Surface_1* (4.99%) was the most dominant family (Fig. 2b), followed by unclassified *SAR11* (1.39%), *Deep_1* (0.43%), *Surface_4* (0.32%), and negligible *Surface_3* (0.001%). The *AEGEAN-169_marine_group* was the single dominant group of *Rhodospirillaceae*, comprising 4.55% of total reads and ~73% of *Rhodospirillaceae* reads. 97% of *Euryarchaeota* reads were assigned to the *Marine Group II* (MG-II) *Euryarchaeota* (24.33% of total reads) compared to 0.73% of all reads assigned to *Marine Group III* (MG-III) *Euryarchaeota* (Fig. 2b). *Chloroplasts* (6.82%), *Synechococcus/Prochlorococcus* (1.44% *Synechococcus* and 0.85% *Prochlorococcus*) and *ML635J-21* (0.97%) were classified within the *Cyanobacteria* (Fig. 2b). ML635J-21 was placed inside *Cyanobacteria* according to 16S sequence alignments in SILVA but there is no sequenced genome available from this clade, which might well belong to a non-photosynthetic sister group^34^. *Bacteroidetes* (8.17%) was the fourth most abundant phylum with mainly *Flavobacteriaceae* (5.01%) and *NS9_marine_group* (2.46%). *NS4_marine_group* (3.37%) and *Sufflavibacter* (1.39%) were the most abundant groups within the *Flavobacteriaceae*.

We may have underestimated a fraction of these microbes as our samples were obtained using 0.45 μm pore size filters. *Candidatus* Pelagibacter (average size 0.2 μm × 0.4 μm) or the tiny *Candidatus* Actinomarina minuta^35^ may have been lost at the beginning of filtering and only retained when other cells already clogged the filters. However, the principally identified taxa are consistent with previous observations using CARD-FISH and 16S rDNA profiling, which also showed that *Archaea* make up to 20% of the prokaryotic community in the Gulf^36^. The low frequency of cyanobacteria appears consistent with seasonal phytoplankton dynamics in the Gulf with cyanobacteria comprising <5% and eukaryotic algae comprise 85–95% of the total chlorophyll *a* when water-column mixing is maximal^20^,^29^,^33^.

The high percentage of *Proteobacteria* we observed is consistent with previous studies from the Red Sea including analyses from the TARA Ocean microbiome^37^. However, relative abundances of *Euryarchaeota* ranged from 1.94% to 7.82% in the TARA Ocean microbiome study^37^, and mainly consisted of *Halobacteriales.* The dominance of *Halobacteriales* within the *Archaea* in the upper layers of the Red Sea was cross-validated by a study of vertical stratification of microbial communities in the Red Sea^38^. During vertical stratification, *Desulfurococcales (Crenarchaeota*) were the dominant archaeal group in the deeper layer^38^. Vertical circulation patterns during the deep mixing period are probably the principal drivers elevating archaeal populations to the surface photic layer. However, it was unexpected that we found MG-II *Euryarchaeota* as the most dominant archaeal group in the surface waters and not *Halobacteriales* or *Desulfurococcales*.

### Global comparison of metatranscriptomes: mdRNA-Seq versus RNA-Seq

**Figure 1:**
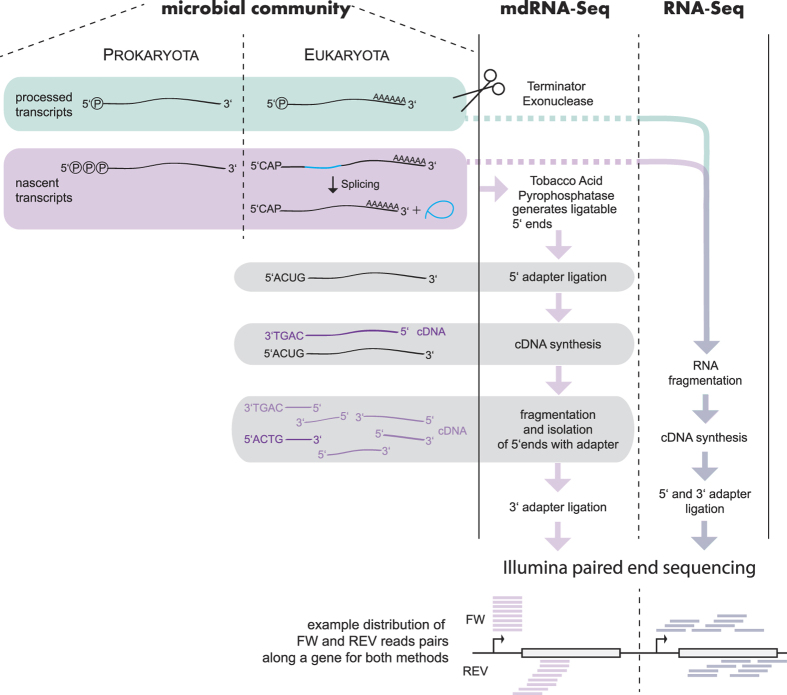
Comparative overview of the transcript library preparation for mdRNA-Seq and RNA-Seq from the microbial community. Seawater was pre-filtered in the shade through a 20 μm mesh to keep out larger material and then onto polyethersulfone filters of 0.45 μm pore size. After isolation of total RNA, RNase treatment and cDNA synthesis, the samples were paired-end sequenced on an Illumina HiSeq 2000 platform with a read-length of 100 nt. An example of the theoretical read distribution over a protein-coding gene and its 5′ UTR is given at the bottom. The arrows mark the TSSs. Only steps important for understanding are sketched; cDNA amplification steps were omitted. 5′ adapter sequences are indicated as “ACUG”.

**Figure 2:**
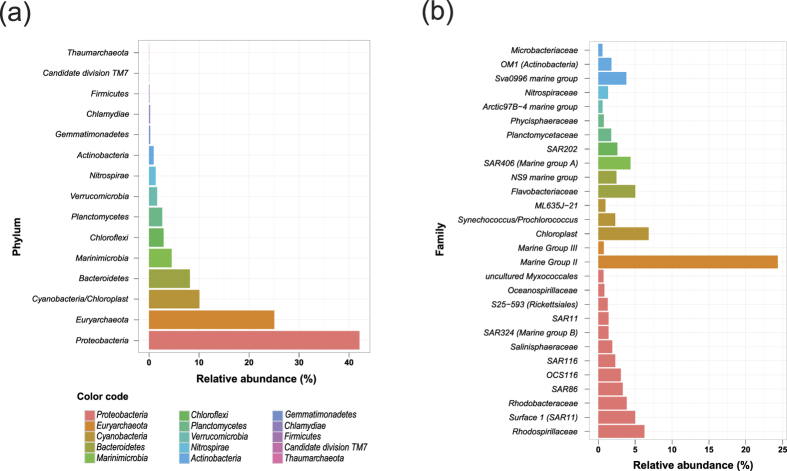
Relative abundance of microbial taxa determined by 16S amplicon sequencing. Abundances are reported as percent of the total count of merged MiSeq reads. 16S sequences from chloroplasts of eukaryotic phytoplankton were not classified further (SILVA pipeline default). **(a)** Taxa abundances at phylum level. Only taxa with > 0.01% abundance were considered. **(b)** Abundances at family or the next available level. Only taxa with > 0.5% abundance were included.

**Figure 3:**
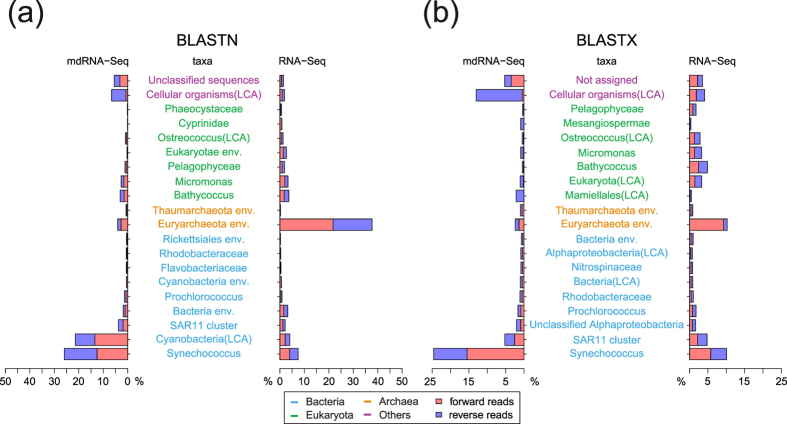
Taxonomic assignments of metatranscriptomic reads. FW (red rectangles) and RV (blue rectangles) reads of the different libraries were compared against NCBI nucleotide (nt) and protein (nr) databases using BLASTN **(a)** or BLASTX **(b)**. The graphs are separated into eukaryotic (green), archaeal (orange), bacterial (blue) taxa, and other nodes (purple). The top-20 ranked taxonomic bins are shown as the percentage of total reads with a BLAST hit (sum of FW and RV reads). “Cellular organisms” is a node specification in NCBI taxonomy for all cellular life including *Archaea*, *Bacteria* and *Eukaryota*. The suffix “LCA” denotes that the reads were assigned to these nodes by the latest common ancestor algorithm, which moves reads with ambiguous assignments higher up in the phylogeny.

For mdRNA-Seq, 53% of the reads (both orientations) yielded matches to sequences in the NCBI nt (nucleotide) database, whereas this was true for only 39% of the forward (FW) and 33% of the reverse (RV) RNA-Seq reads (Table 1). The low hit rate for both approaches is consistent with the high sequence diversity within marine microbial communities^39^. We also found a substantial difference in the specificity of the identified taxa (Table 1, Fig. 3). mdRNA-Seq identified approximately 3.7 times more bacterial reads than RNA-Seq; for cyanobacteria, this difference was even higher with 5.7 times more assigned reads in mdRNA-Seq. Conversely, we found approximately 4.5 times more archaeal reads in the RNA-Seq than in the mdRNA-Seq libraries. Overall, such differences might lead to different conclusions from the sequenced metatranscriptome, namely, the dominance of cyanobacterial transcripts with mdRNA-Seq and the dominance of euryarchaeal transcripts with RNA-Seq (Fig. 3). However, the high representation of *Euryarchaeota* in the RNA-Seq dataset was linked to 13 mRNAs contributing 93% of euryarchaeal RNA-Seq reads, thus suggesting that the mdRNA-Seq analysis may be more realistic. When we compared the datasets to the NCBI nr (non-redundant protein) database, mdRNA-Seq yielded more than twice as many hits as RNA-Seq (Table 1). While in the RNA-Seq dataset, FW and RV reads matched in similar proportions (14.5% and 12.7%, respectively), this was not the case for the mdRNA-Seq dataset. Here, the database covered a larger fraction of RV reads than FW reads (39.4% and 24.5%, respectively; Table 1). This was especially evident for eukaryotic reads, of which only 1.6% of FW, but 7.3% of RV reads were assigned. These findings underscore the conclusions that the two library preparation techniques lead to specific differences. The difference in the ratio between database coverage for FW and RV reads reflects the mdRNA-Seq focus on the 5′ ends of transcripts, as most genes are transcribed with 5′ untranslated regions (5′UTRs), and mdRNA-Seq FW reads capture these 5′UTRs (Fig. 1). Depending on 5′UTR length, only a fraction of the 100 nt Illumina FW reads extend into the coding sequence. The resulting partial matches may not suffice for a BLASTX hit. Accordingly, the particular underrepresentation of mapped eukaryotic mdRNA-Seq FW reads reflects the generally longer 5′UTRs of eukaryotes compared to bacteria. Conversely, RNA-Seq FW reads are distributed over the whole length of transcripts, thus generating multiple hits against the coding sequence. Therefore, mdRNA-Seq will miss genes transcribed within operons. However, depending on the insert size chosen for sequencing (here: 300–500 nt), the RV reads for both library types should be predominantly located within coding regions.

The mdRNA-Seq approach we applied here worked on eukaryotic as well as bacterial and archaeal transcripts alike. However, the largest portion of additionally identified reads in the mdRNA-Seq dataset belonged to bacteria. The higher database hit rate - comparing nucleotide versus protein sequences - reflects the strength of mdRNA-Seq in the identification of non-coding transcripts. We conclude that mdRNA-Seq is more efficient when producing reads for bacterial transcripts. With its focus on nascent transcripts, mdRNA-Seq is closer to the actual transcriptional (promoter) activity and likely more suitable to illuminate differentially expressed genes.

### Metatranscriptome mapping to representative species

For the identification of representative genomic sequences, the *in-situ* transcriptomes were compared against the NCBI databases and the results were imported into MEGAN v5.2.0^40^ for global taxonomic assignments as shown in Fig. 3. Taxa with particularly high coverage were “*Synechococcus*”, the “*SAR11 cluster*/*Alphaproteobacteria*”, “*Archaea*” and “*Mamiellales*”. To find a suitable reference genome for each of these taxa, the corresponding sets of reads were aligned to all complete and draft genomes available for these clades at different identity cut-offs (Figure S1 and Supplementary Methods: *Model reference genome selection*).

For this we selected genome sequences recruiting a maximum of reads. The selected references were strain *Synechococcus* sp. CC9605 for “*Synechococcus”* and *Candidatus* Pelagibacter sp. HTCC7211 for the “SAR11/*Alphaproteobacteria*” cluster.

*Synechococcus* CC9605 was isolated from the California current and rigorously characterized^41^,^42^. It belongs to clade II within the picophytoplankton subcluster 5.1A^41^, which is typical for offshore oligotrophic subtropical and tropical waters between 30°N and 30°S^43^. *Candidatus* Pelagibacter sp. HTCC7211 is a representative of subclade Ia (open-ocean, subtropical)^44^, which accounted for the majority (25.5%) of RNA-Seq reads mapping to SAR11-type bacteria (Figure S1B).

We chose the chromosome, mitochondrial and chloroplast sequences of the picoeukaryotic alga *Micromonas* RCC299^45^ to represent *Mamiellales* (genera *Bathycoccus, Micromonas* and *Osterococcus*), which made up 44.9%/33.5% FW and RV reads assigned to *Mamiellales* (Fig. 3). Sequences classified as “environmental samples <Euryarchaeota>” recruited 97.7%/96.3% of RNA-Seq and 77.9%/63.9% of the mdRNA-Seq FW/RV reads assigned to *Archaea*. At the outset of our study, only one composite genome was available for the marine *Euryarchaea* MG-II^46^ and several *Thaumarchaeota* reference genomes, for example, *Candidatus* Nitrosopelagicus brevis^47^ or *Nitrosopumilus maritimus*^48^. However, for the reads in this study the metagenomic fosmids and assemblages^49^,^50^ turned out as the best reference, especially the contigs of uncultured MG-II/III *Euryarchaeota*, originating from a mediterranean metagenomic library with 997 contigs in total^50^. The “Archaea” reads were aligned to these contigs and 201 contigs selected that each recruited at least 100 reads. From these, 149 contigs were ascribed to MG-II/III *Euryarchaeota* (yielding a total of 5.4 Mb), and 52 contigs were classified as *Thaumarchaeota* (total of 1.9 Mb). For all representative species, we provide genome-wide graphical overviews of all mapped RNA-Seq and mdRNA-Seq reads for further exploratory work (Datasets S1–S5) as well as the details and classification of all predicted active TSSs (Tables S2, S4–S6).

### Transcriptome mapping of environmental reads to COG categories highlights major metabolic processes

We analyzed all transcriptome data mapping to the selected genome sequences (including archaeal contigs) with regard to the most important metabolic processes in these organisms. For both libraries, we summed the number of FW/RV reads mapping to the 25 COG functional categories (Figure S4). The share of reads assigned to COG was highest (>96%) for *Candidatus* Pelagibacter sp. HTCC7211, in line with its relatively well-annotated, streamlined genome, intermediate for *Synechococcus* and *Micromonas* and very low for MG-II contigs.

For both the *Cyanobacteria* and *Micromonas* reads, the principal functional categories were *“Translation, ribosomal structure and biogenesis”* and “*Energy production and conversion”.* “*Carbohydrate transport and metabolism”* was another highly represented category. “*Posttranslational modification, protein turnover, and chaperones* were also categorized as highly active processes in all organisms. For *Candidatus* Pelagibacter sp. HTCC7211 the major COGs belonged to “*Amino acid transport and metabolism”.* For *Thaumarchaeota* the predominant category was “*Inorganic ion transport and metabolism”* (Figure S4). “*Chromatin structure and dynamics”* and “*RNA processing and modification”* were specifically enriched in the eukaryotic *Micromonas,* along with its extensive intron processing machinery^51^, setting it apart from the other organisms. Furthermore, mapping of mdRNA-Seq reads to *Micromonas* revealed 21 TSSs in the mitochondrial genome, 13 in the chloroplast genome and 1,742 in the 17 chromosomes of the *Micromonas* strain RCC299 (Table S4, Dataset S3). This demonstrates that mdRNA-Seq provides precise information on the promoters used in the eukaryotic nucleus as well as in mitochondria and chloroplasts. Of the 38 genes accounting for the most reads in *Micromonas*, three were mitochondrial and 12 chloroplast-located. These top-expressed genes encode proteins with predominantly energy-related functions throughout all three genetic compartments of the cell, consistent with the high ranking of the “Energy production and conversion” COG category (Figure S4).

The COG classifications were generally similar for the two different datasets despite the differences in absolute read assignments.

### The primary *in-situ* transcriptome of Synechococcus CC9605

The inferred, comprehensive *in-situ* primary transcriptome provides information on the actual transcriptional activity of individual promoters in an organism in its natural environment. Further information is gained on putative non-coding RNAs (ncRNAs) and antisense RNAs (asRNAs), which are often not considered in metatranscriptome analyses. For *Synechococcus* CC9605, we inferred 1005 TSSs transcribing annotated genes (gTSS), 58 TSSs transcribing ncRNAs (nTSS), 175 TSSs within coding sequences (iTSS) and 127 aTSSs transcribing asRNAs (Table S2, Dataset S1). This corresponds to 37% of all protein-coding genes detected as associated with an active TSS *in situ*, compared to 29% of all genes covered by RNA-Seq (requiring the same minimum coverage of 13 reads). This percentage of 37% is only slightly less than that recovered in the primary transcriptome analysis of the marine picocyanobacteria *Prochlorococcus* MED4 and MIT9313 from laboratory cultures (49 and 41%, respectively)^10^, and underscores the suitability of mdRNA-Seq for the analysis of microbial communities. Moreover, the mdRNA-Seq information enabled the identification of genes for 44 novel proteins in the genome sequence of *Synechococcus* CC9605 and the corrected annotation of 86 protein-coding genes (Table S3).

Of the 50 top-recruiting TSSs in *Synechococcus* CC9605, 43 were associated with protein-coding genes (Table S2). Of these, 16 encode proteins involved in photosynthesis, and three encode enzymes involved in tetrapyrrole biosynthesis, consistent with the importance of oxygenic photosynthesis for these organisms. Another nine encode proteins involved in protein biosynthesis, one encodes an outer membrane porin, one an RNA binding protein, and seven genes encode hypothetical proteins. No previous annotations exist for four of the genes associated with highly active TSSs. Based on conservation of the deduced amino acid sequence we identified one of them as encoding Ycf12 (Psb30), a core subunit of photosystem II^52^. The others, here called Syn_n046, Syn_n048 and Syn_n060, are peptides of 54, 41 and 52 amino acids, respectively (Table S3). Homologs of Syn_n060 were detected in many genome sequences of marine bacteriophages and picocyanobacteria, including the different major ecotypes of *Prochlorococcus.* The fact that this gene family is widely distributed and highly expressed suggests that it plays an important role in the marine environment. Here, the transcriptomic data provide clear-cut evidence not only of their expression but also of their association with a distinct TSS.

The highest-scoring TSS for a protein-coding gene was associated with the *petF-3* gene (*Syncc9605_2148*), which encodes the major ferredoxin I. There are 6 different ferredoxin genes in *Synechococcus* CC9605, a sequence comparison against the well-studied *Synechocystis* sp. PCC 6803 shows *ssl0020* as the top-scoring match, reinforcing the gene product as ferredoxin I. This form is the most abundant ferredoxin in photosynthetic organisms, mediating multiple redox processes. These include electron transfer from PSI to ferredoxin-NADP-reductase^53^ that reduces NADP^+^ for CO_2_ fixation, sulphite reduction, fatty acid metabolism and activity modulation of various enzymes via the thioredoxin system^54^, strongly underscoring the relevance of photosynthetic electron transport for these organisms.

**Figure 4:**
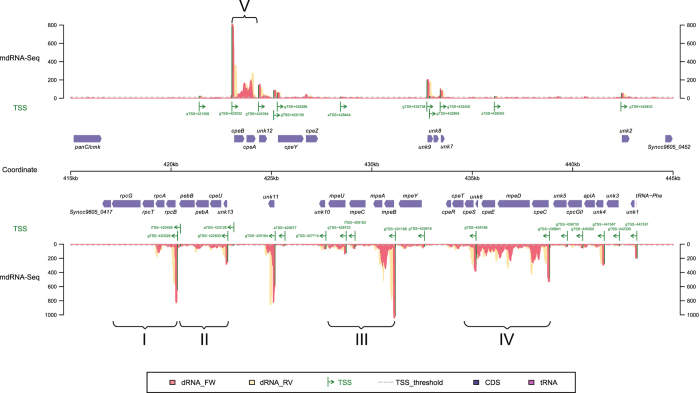
The *Synechococcus* CC9605 phycobiliprotein gene cluster with 32 identified TSSs (indicated by green arrows) inferred from mdRNA-Seq coverage. The TSS upstream of *mpeB* is amongst the most active promoters overall (position 59 in Table S1). There are 38 annotated genes in this region^41^,^42^, of which 13 are of unknown function (*unk1* – *unk13*). Roman numerals I to V indicate 5 major transcriptional units that encompass almost all protein-coding genes with a known function and that are associated with very strong TSSs. There is one additional TSS recruiting a similar number of mdRNA-Seq reads, mapping to position 425184r, driving the transcription of *unk11* a gene of unknown function^42^. The coverage resulting from reads originating from mdRNA-Seq in FW orientation are shown in light red and the respective associated reads in RV orientation in yellow. Additional details, including the mapped RNA-Seq coverage can be found in Supplemental Dataset S1.

A remarkable set of active promoters was associated with the genes for phycobilisomes, chromophore and phycobiliprotein synthesis. In marine S*ynechococcus,* these genes are frequently organized in the form of distinct gene clusters^41^,^55^, some of which are up to 29 Kbp in size. *Synechococcus* CC9605 harbors a PBS gene cluster that encompasses at least 38 genes and constitutes a genomic island^41^,^42^. We mapped 15 major active TSS to this gene cluster (Fig. 4). The most active promoters in this region were associated with the *cpeBA* and the *mpeBA* genes encoding the alpha and beta subunits of the two phycoerythrin types, the *rpcBA* genes encoding the two phycocyanin subunits and the gTSS at 425184 constituting the TSS of the short un-annotated gene *unk11*^42^, underlining its likely important function (Fig. 4). We also detected transcript coverage for all other genes of unknown function (*unk*s), collectively underlining their likely important functions in photosynthesis of natural *Synechococcus* populations.

Furthermore, we noticed an antisense RNA IsrR-1b for gene *Syncc9605_1590 (pcbD*), which encodes a protein similar to the light-harvesting proteins in *Prochlorococcus* (Dataset S1). This situation resembles the *isiA* gene in *Synechocystis* 6803, which is post-transcriptionally controlled by the asRNA IsrR^56^ and related at the protein level (56% identical, 71% similar residues). Thus far, homologs of IsrR have only been detected in the two model species *Synechocystis* 6803 and 6714^56^,^57^. We detected the asRNA to *pcbD* but not the mRNA, indicating a possible co-degradation mechanism similar to the *isiA*/IsrR transcript pair in *Synechocystis*^56^. Therefore, this result suggests a wider occurrence of this regulatory mechanism. The fact that *Syncc9605_1590* was not expressed in our dataset is consistent with the fact that the Gulf is likely not limited by iron^58^.

Non-coding RNAs can play important regulatory roles, e.g., in cyanobacteria in the adaptation of the photosynthetic apparatus to high light intensities^59^ or of the nitrogen assimilatory machinery to nitrogen limitation^60^. In addition to protein-coding genes, the metatranscriptome mapping to *Synechococcus* CC9605 revealed the existence of more than 200 *in-situ* transcribed ncRNAs and asRNAs. Seven of these ncRNAs were paralogs of each other ranging from 290 to 328 nt, and together represented the most highly accumulating transcript species (Figure S3). All seven paralogs combined accounted for 55.2% of *Synechococcus* CC9605 mdRNA-Seq reads. Homologs of this ncRNA exist in several marine *Synechococcus*, including strains WH8109, WH7803, CC9311, KORDI-49, WH8102, KORDI-52, KORDI-100, RCC307 and CC9902, as well as in *Prochlorococcus* CCMP1375 (SS120), NATL1A and 2A, MIT9303 and MIT9313. Based on these homologs, we predicted a strongly conserved secondary structure, which is a typical feature of many ncRNAs (Figure S3A). Sequence comparison and phylogenetic analysis revealed a *Synechococcus-* and a *Prochlorococcus*-specific clade (Figure S3B). Indeed, Yfr103 was the single most abundant ncRNA in laboratory cultures of *Prochlorococcus* MIT9313^10^. In these laboratory experiments, Yfr103 responded positively to fluctuating light and temperature conditions, suggesting its possible involvement in light or temperature acclimation. Moreover, Yfr103 has been the single most abundant transcript within environmental populations of marine picocyanobacteria in the Southwest Pacific (Pfreundt *et al.*^61^). Here, we show that Yfr103 is very abundant in native microbial communities from the Gulf at the time of sampling. Finally, we observed a conserved imperfect palindromic sequence in the region from −29 to −58 ±1 nt with regard to the mapped TSS, which might be a transcription factor binding site (Figure S3C). Whereas the function of Yfr103 remains enigmatic, its high expression, wide distribution and copy number are compatible with a central structural or regulatory function.

### High expression of the glycine riboswitch in SAR11 type Alphaproteobacteria

The list of active promoters mapped to the genome of *Candidatus* Pelagibacter sp. HTCC7211 (Dataset S2), representing SAR11-type *Alphaproteobacteria*, was dominated by transport functions, especially driving the transcription of genes for the detection and transport of taurine, spermidine/putrescine, ammonium or trimethylamine N-oxide, a common osmolyte^62^ in marine biota (Table 2). The bacteriorhodopsin gene was highly transcribed (ranked 10^th^ among the TSSs), in accordance with its function as a light-driven proton pump, generating biochemical energy from light^63^,^64^.

The TSS associated with *glcB* drew special attention as it is located 162 nt upstream of the start codon contrasting with fact that these bacteria have the shortest intergenic spacers ever observed^65^. The *glcB* gene encodes for the malate synthase protein, an essential enzyme for these bacteria, as it enables the regeneration of TCA intermediates through the glyoxylate cycle^66^. *glcB* is controlled by the glycine riboswitch linking the intracellular glycine level to the regulation of carbon partitioning for biosynthesis or energy^66^. This mechanism is exclusive for SAR11 and thought to result from the extensive genome streamlining^66^. Detailed analyses of the mdRNA-Seq raw reads mapping to this locus showed that indeed these transcripts constituted the riboswitch for glycine. The respective reads started directly with the first nucleotide of the glycine riboswitch P1 domain (Figure S5), or in some cases, started up to 15 nt upstream. To further check the downstream genes linked to this riboswitch in our sample, we assembled the recruited paired reads. The assemblies showed that these reads actually linked to *gcvT*, the glycine cleavage system T protein, which was the second gene controlled by a glycine riboswitch in the reference genome. Thus, *gcvT* was more highly expressed than *glcB* under the sampled environmental conditions. We found no further genes linked to this riboswitch. Our finding that *in situ,* transcripts of the glycine riboswitch were mainly extending into the gene *gcvT* suggests that the cells must have been glycine-replete.

### Transcriptional activity of abundant marine Euryarchaeota

Metagenomic scaffolds for MG-II *Euryarchaeota*^49^,^67^ accounted for the largest proportion of reads assigned to *Archaea* (Dataset S4, Table S5). Eight of the 149 selected contigs stood out accounting for 92% of the RNA-Seq reads (Figure S1C) and 45% of the mdRNA-Seq reads mapping to euryarchaeal sequences. Nevertheless, we detected substantial transcription linked to many genes distributed over all 149 contigs, supporting the view that these contigs represented *Euryarchaeota* present at the time of sampling in the Gulf reasonably. It is worth noting that the MG-II fosmids used here for interpretation came from very deep waters (1000–3000 m) in the Mediterranean^49^ in comparison with the analyzed sample from 45 m. This fact indicates the existence of related *Euryarchaeota* in the Red Sea. These may normally thrive in deeper waters and might have been found here close to surface due to the deep mixing.

Only 12 of the 30 euryarchaeal protein-coding genes with highest read numbers encode a known protein or protein domain, and three of them are involved in gene expression (protein biosynthesis: RP-L12, elongation factor EF-1 subunit alpha; transcription: TFIID). The seventh-most abundant TSS was associated with gene *eury_gene80 (GI:663535065)*, which encodes a DUF1628/COG3430 domain containing archaeal flagellin (archaellin); this domain was characterized as the N terminus of archaeal PilA adhesion pilins^68^. *livK as* the tenth-most abundant TSS in the *Euryarchaeota* contigs (Table S5) suggests that this periplasmic substrate-binding protein of the branched amino acid transporter family plays an important function and corresponds to previous suggestions that MG-II can utilize organic carbon in the surface oceans^47^,^69^.

Several genes encoding enzymes in the synthesis of important co-factors were highly expressed as well, such as pyridoxal (vitamin B_6_) biosynthesis (*pdxS*, pyridoxal 5′-phosphate synthase), riboflavin (vitamin B_2_) synthesis (*ribA* and *ribB*, GTP cyclohydrolase II and 3,4-dihydroxy 2-butanone 4-phosphate synthase), or in the degradation of aromatic compounds such as benzoyl-CoA oxygenase (*boxB*). It is well known that some vitamins (especially B_1_, B_7_, and B_12_) within marine microbial communities originate from the activities of only certain bacteria and algae, whereas others are opportunistic or even auxotrophic^70^. Therefore, these results point to hitherto unknown functions of Euryarchaea within their ambient microbial communities and suggest that provision of vitamins could be among them.

The greatest share (93%) of RNA-Seq reads recruited by the euryarchaeal contigs originated from only 13 mRNAs (Table S5). This essentially accounted for the much higher mapping percentage of RNA-Seq vs mdRNA-Seq reads to Archaea (Table 1). Searching for commonalities among the encoded proteins, we found that all 13 proteins belong to the class of intrinsically disordered proteins (IDPs) or contain disordered regions. IDPs are unable to fold spontaneously into stable three-dimensional structures; instead, they fluctuate dynamically through a range of conformations. This property frequently makes them an important component within signaling networks, as the same polypeptide can perform different interactions, leading to different results^71^. Nine of the 13 IDPs form two clusters of sequence homologs (Figure S6), from which one was annotated as “putative collagen-like cell surface protein” in one MG-II metagenome assembly^46^ and also exists in *Candidatus* Thalassoarchaea^49^ (Figure S6B). Some *Archaea* are rich in IDPs^72^, but to our knowledge this is the first report that IDPs are exceptionally highly expressed in *Euryarchaeota*.

**Figure 5:**
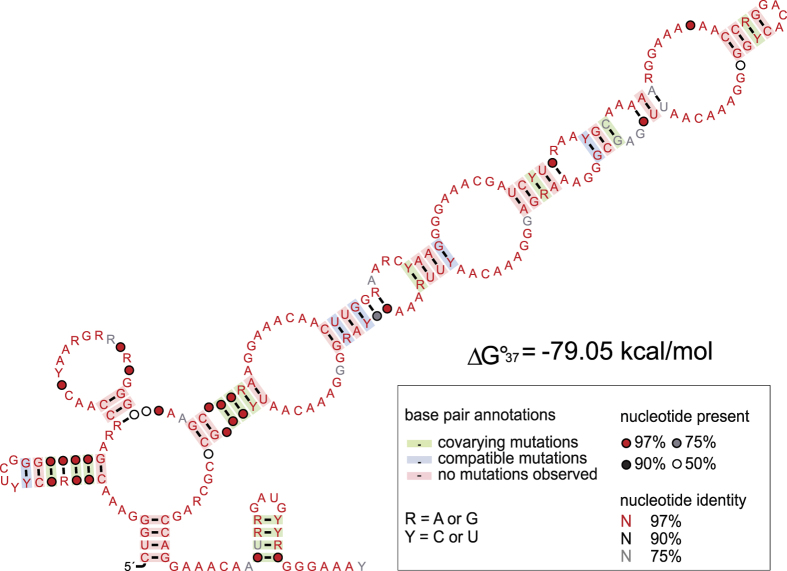
Conserved secondary structure of the *Candidatus* Thalassoarchaea ncRNA 1 (Tan1).

Additionally, we identified several TSSs associated with non-coding regions in *Euryarchaeota* (Table S5). Nine abundant, slightly divergent ncRNAs with lengths between 292 and 306 nt mapped in antisense orientation to an annotated gene. Searches against the RFAM database revealed that these sequences matched RNaseP_arch (accession RF00373), i.e., they constitute the RNA component of RNase P. To verify this possibility, we added the nine sequences to the seed alignment (Dataset S5) and found conserved essential structural components of RNase P RNA (Figure S7). Thus, the gene annotated on the complementary strand is likely a misannotation. In a similar fashion, we identified another type of 305–315 nt long ncRNA to represent the RNA of the signal recognition particle (Archaea_SRP, RF01857). In addition, we identified an unknown ncRNA of 229 nt ranked 5^th^ in the RNA-Seq analysis (Table S5). BLASTN searches against NCBI nt database identified five homologs with identity ranging from 75 to 79%, all belonging to the metagenomes of *Candidatus* Thalassoarchaea^49^. Here, we denote this ncRNA as ***T**halasso**a**rchaea* ncRNA 1 (Tan1) and suggest a complex secondary structure based on a multiple sequence alignment of the six homologs (Fig. 5).

### Distribution of reads assigned to rhodopsin genes

To assess the phylogenetic distribution of rhodopsin transcripts, we assigned the reverse reads from both the mdRNA-Seq and RNA-Seq libraries to the reference rhodopsin sequences of the Red Sea rhodopsin diversity study^30^. Ambiguous reads were assigned to their common ancestors using MEGAN^40^. Group I proteorhodopsin env142271822_2 (recovered from marine metagenome)^73^ recruited 14.96% of all assigned rhodopsin reads, which was the top number (Figure S9). Two SAR11-type group I proteorhodopsins followed, encoded by the genes GYQIE2T01DYL4Q (7.80%) and GYQIE2T01BHUTZ (7.20%)^30^. Other sequences recruiting more than 3% of the rhodopsin reads included ENV142932331_2 (5.74%) and SAR11_pelagibacter_HTCC7211 (3.31%). However, the highest share, 30.79% of all rhodopsin reads, could not be assigned uniquely and was assigned to the common ancestor of GYQIE2T01BHUTZ and SAR11_pelagibacter_HTCC7211. Altogether, 43.39% of all rhodopsin reads came from SAR11-type proteorhodopsins (Figure S9). In total, MG-II *Euryarchaeota* recruited 10.05% of all rhodopsin reads. From these, two euryarchaeal rhodopsin sequences, group_II_euryarchaeote_HF70_59C08 and group_II_euryarchaeote_HF10_3D09, recruited 0.26% and 0.62% of all rhodopsin reads, respectively (Figure S9).

### Highly expressed peptidases and carbohydrate-active enzymes

**Figure 6:**
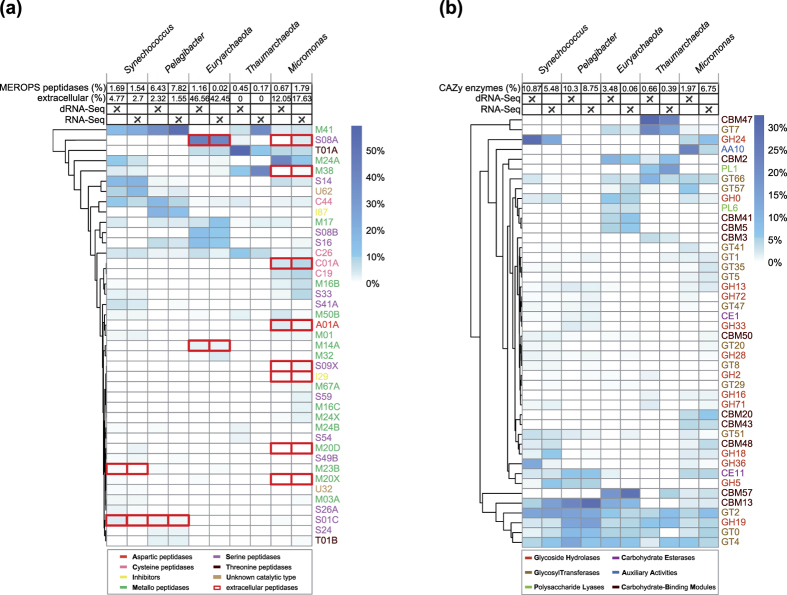
Transcriptome share of genes related to peptidases and carbohydrate-active enzymes. The heat maps show the relative distribution of reads assigned to MEROPS^75^ peptidase families **(a)** and to CAZy^76^ carbohydrate-active enzyme classes/modules **(b)**. Read counts were normalized by total number of reads assigned to each reference for each library. Only families or classes/modules with a minimum relative read count of 2% when added up across all references are shown. Rows were hierarchically clustered using the complete-linkage method according to pairwise euclidean distances. The percentages denote the fractions of reads assigned to MEROPS^75^ peptidases or CAZy^76^ enzymes, compared to all reads assigned to the corresponding reference. Families representing extracellular peptidases are highlighted by red rectangles. The percentages of reads assigned to extracellular peptidases compared to reads recruited by MEROPS peptidases are given on top.

Utilizing extracellular peptidases and carbohydrate-active enzymes, MG-II may assimilate organic carbon and nitrogen from the surface oceans^47^,^69^. This corresponds with our finding of highly expressed amino acid transporters (Table S5). Li *et al*. (2015) introduced a scheme for the classification of genes encoding such extracellular peptidases and carbohydrate-active enzymes^74^. We mapped metatranscriptome reads against peptidase families in the database MEROPS^75^ and against the CAZy^76^ carbohydrate-active enzyme classes focusing on our 5 reference genomes (Fig. 6). More than 40% of the reads assigned to peptidases in MG-II *Euryarchaeota* were predicted to be extracellular. Especially, the enzyme families S08A (43.64% for mdRNA-Seq and 40.25% for RNA-Seq) and S08B (14.78% for mdRNA-Seq and 10.69 for RNA-Seq) recruited many reads. S08A was previously reported as the dominating extracellular peptidase of MG-I, II, and III, suggesting its important role in the assimilation of nutrients by extracellular protein degradation^74^. *Micromonas* held with more than 10% a relatively high share in the expression of extracellular peptidases, too. Some expression of such enzymes was detected also for *Synechococcus* and *Pelagibacter*. In case of the latter this is consistent with the lack to synthesize the amino acids glycine and serine^66^ and the fact that amino acid transporters were among the most highly expressed genes (Table 2). In contrast, none of the expressed peptidases in *Thaumarchaeota* was predicted to be extracellular (Fig. 6). The investigated reference genomes also contained multiple expressed carbohydrate-active enzymes (Fig. 6). Here, *Thaumarchaeota* were very active, suggesting their use of extracellular carbohydrates as a source of carbon. Furthermore, interesting parallels were found between *Synechococcus*, Pelagibacter and MG-II (bottom part of Fig. 6). These results point to heterotrophic or mixotrophic elements in the cyanobacteria and microalgae, which fits independent observations^77^,^78^ and suggest parallels as well as decisive differences in the lifestyles of the here investigated microbes.

## Conclusion

Readily applied on cultured bacteria, archaea and human cells^7^,^8^,^9^,^10^,^11^,^79^, dRNA-Seq can produce precise primary transcriptomes. These enhance differential expression analysis by focusing on nascent transcripts, revealing new promoter motifs, previously unknown ncRNAs, and asRNAs. An advantage of transferring this approach to metatranscriptomic analysis (mdRNA-Seq) is the possibility of extracting information on the organization of primary transcriptomes of a natural microbial community *in situ.* As demonstrated here, mdRNA-Seq may be most valuable when reference genomes closely related to those of target taxa are available. Although in many systems reference genomes are still lacking for certain taxa, advances in new cultivation approaches and sequencing technologies are reducing the gaps. Alternatively, mdRNA-Seq may be combined with metagenome assembly, creating information about promoter activities in abundant organisms completely *de-novo*. Here, we specifically employed the genome sequences of *Synechococcus* CC9605, *Candidatus* Pelagibacter sp. HTCC7211, the three genetic compartments of *Micromonas* sp. RCC299, and environmental sequences for marine type II/III *Euryarchaeota* and *Thaumarchaeota* to elucidate the efficiency of primary transcriptome recovery from a complex environmental sample. These organisms correspond to a substantial share of the reads in the analyzed metatranscriptomes. The enzymes used for mdRNA-Seq library preparation work equally on both the 5′ CAP and 5′ PPP (i.e., both eukaryotic and prokaryotic nascent transcripts are selectively sequenced). This was illustrated by primary transcriptome data obtained for transcripts encoded in the nucleus, the chloroplast and the mitochondrion of a eukaryotic alga, in bacteria and in archaea alike.

Inferred information from these analyses includes the precise identification of TSSs, riboswitches and conserved promoter motifs, median 5′ UTR lengths, and cases of nested transcription. Detection is further facilitated for transcripts originating from promoters transcribing unknown genetic elements, such as ncRNAs, asRNAs, and gene-internally transcribed RNAs (iTSS). This is a complicated task using conventional RNA-Seq, where transcription in non-coding regions is difficult to distinguish from noise as there is no clear 5′ end transcript detectable. Conventional RNA-Seq also prevents the detection of iTSS as they will be counted as transcription of the gene itself. Therefore, unless ncRNAs are specifically targeted^1^, metatranscriptomic analyses ignore these transcripts.

We show that the mdRNA-Seq approach is feasible for highly complex microbial samples.

## Methods

### Sampling of marine microbes, cDNA preparation and sequence analysis

Samples were taken in the Gulf of Aqaba (29°28′N 34°55′E, ~700 m bottom depth), from a depth of 45 m on May 02, 2012, between 9:45 and 14:45 (GMT + 2). From each of four Niskin bottles, 10 L of water was collected and immediately pre-filtered in the shade through a 20 μm mesh to keep out larger material and then onto polyethersulfone filters (PALL Supor, 47 mm diameter, 0.45 μm pore size). The maximal filtration time was 20 minutes. Filters were placed in 1 ml of RNA resuspension buffer (10 mM NaAc pH 5.2, 200 mM D(+)-sucrose, 100 mM NaCl, 5-mM EDTA), immediately frozen in liquid nitrogen, and kept at −80 °C until further analysis. Total RNA from each of the 4 filters (i.e., 4 Niskin bottles) was pooled after extraction. For mdRNA-seq, total RNA was treated with 5′-Terminator Exonuclease (Epicentre), which degrades processed transcripts. To block possibly remaining 5′-monophosphate ends, an oligonucleotide was ligated to the treated RNA (not shown). Tobacco Acid Pyrophosphatase (Epicentre) then generated ligatable 5′ ends from the remaining nascent (protected) transcripts, followed by 5′ RNA-adapter ligation and random primed first-strand cDNA synthesis using an N6 randomized primer and M-MLV reverse transcriptase (New England Biolabs). Then, an Illumina adapter was ligated to the 5′ ends of the antisense cDNA and the subsequent PCR (second-strand cDNA synthesis and 16 PCR cycles, not shown) added a biotin-tag to the original 5′ ends. After fragmentation of the cDNA with three 30 s ultrasound pulses at 4 °C, these 5′ fragments were isolated with streptavidin beads, and a second Illumina adapter was ligated to the 3′ ends, followed by another 5 PCR cycles. For RNA-Seq, the RNA was directly fragmented, reverse-transcribed using random hexamers, and Illumina TruSeq-adapters added to both ends. Both cDNA libraries were eluted in the size range of 250–500 bp from a preparative agarose gel and were paired-end sequenced on an Illumina HiSeq 2000 platform generating 2 × 100 bp reads. Additional details are given in the legend to Fig. 1. All raw reads can be downloaded from the NCBI Sequence Read Archive under the BioProject accession number PRJNA248420. The major bioinformatics workflow is illustrated in Figure S2. A detailed description of all transcriptome-related bioinformatics analyses is given in the Supporting Information.

### MiSeq analysis of rRNA sequences, OTU clustering and taxonomic classification

Amplicons corresponding to the ribosomal RNA small subunit hypervariable regions 3–4 were amplified directly from the DNA present within the prepared RNA sample using primers S-D-Bact-0341-b-S-17/S-D-Bact-0785-a-A-21^80^ that target bacteria and archaea. The amplicons were sequenced on a MiSeq (Illumina) system with a read length of 2 × 300 nt (paired end), generating 202,556 reads after merging of overlapping paired-end reads. The bioinformatics analysis followed the UPARSE pipeline^81^. Lowest quality tails were truncated from all reads and the paired reads merged using the -fastq_mergepairs command. Merged reads were quality filtered and reads <350 nt were discarded, After conversion to fasta format, all files were concatenated for OTU clustering, with the following steps: dereplication at 100% identity and keeping information on the weight of each unique sequence, sorting of unique sequences by decreasing weight and discarding of singletons and clustering into OTUs with the -cluster_otus command using a maximum dissimilarity of 2% and filtering for chimaeras^81^. Finally, the merged reads were mapped back onto the generated OTUs using vsearch v1.1.3^82^ requiring ≥ 98% identity to create the final OTU table. The resulting list was submitted to SILVA(https://www.arb-silva.de/ngs/) for taxonomic classification using release SSU 119.1^83^. Additional details were described earlier^84^. Supplementary information is also available at https://dx.doi.org/10.6084/m9.figshare.3122254.v1.

## Additional Information

**How to cite this article**: Hou, S. *et al.* mdRNA-Seq analysis of marine microbial communities from the northern Red Sea. *Sci. Rep.* **6**, 35470; doi: 10.1038/srep35470 (2016).

## Supplementary Material

Supplementary Information

Supplementary Tables

## Figures and Tables

**Table 1:** BLASTN and BLASTX results (e-value cutoff 0.00001) for all non-ribosomal FW and RV reads, separately for both libraries.

		Total reads non-rRNA	Total reads with BLASTN/X hits		Bacteria		Eukaryota		Archaea		Viruses		Unclassified	
			Total	%	Total	%	Total	%	Total	%	Total	%	Total	%
BLASTN	mdRNA-seq FW	**6,306,051**	3,339,240	52.95	2,367,277	37.54	458,251	7.27	234,353	3.72	8,876	0.14	214,038	3.39
	mdRNA-seq RV	**6,300,758**	3,346,934	53.12	2,066,011	32.79	574,692	9.12	142,641	2.26	8,288	0.13	156,845	2.49
	RNA-seq FW	**6,306,051**	2,456,294	38.95	651,373	10.33	706,682	11.21	1,006,430	15.96	8,112	0.13	30,928	0.49
	RNA-seq RV	**6,300,758**	2,059,792	32.69	539,054	8.56	750,628	11.91	681,418	10.81	8,324	0.13	32,310	0.51
BLASTX	mdRNA-seq FW	**6,306,051**	1,543,275	24.47	1,180,103	18.71	100,235	1.59	90,212	1.43	4,418	0.07	7,063	0.11
	mdRNA-seq RV	**6,300,758**	2,482,609	39.40	1,197,798	19.01	457,885	7.27	91,261	1.45	7,172	0.11	10,373	0.16
	RNA-seq FW	**6,306,051**	914,685	14.50	313,715	4.97	333,315	5.29	175,119	2.78	5,426	0.09	5,841	0.09
	RNA-seq RV	**6,300,758**	799,880	12.69	304,414	4.83	359,850	5.71	35,419	0.56	7,270	0.12	4,949	0.08

The number of reads and percentage of total non-ribosomal reads is given that were assigned to *Bacteria*, *Eukaryota*, *Archaea* or Viruses.

**Table 2:** Protein-coding genes in *Candidatus* Pelagibacter sp. HTCC7211 recruiting highest read numbers in mdRNA-Seq or RNA-Seq.

Gene name	Locus_tag	Start	End	Strand	TSS	RNA-Seq	Product
potD	PB7211_697	1265830	1266982	−	**1591**	646	spermidine/putrescine-binding periplasmic protein
ybhL	PB7211_203	285807	286571	+	**1034**	234	integral membrane protein
tauA	PB7211_601	704432	705401	−	**917**	440	taurine transport system periplasmic protein
trxB_1	PB7211_981	473324	474270	−	**904**	209	thioredoxin-disulfide reductase
opuAC	PB7211_687	1212766	1213737	−	**828**	1176	substrate-binding region of ABC-type glycine betaine transport system; also called TmoX, was among the 10 most highly expressed genes in metaproteomic analyses of samples collected from the Sargasso Sea^85^.
amt	PB7211_1287	1226344	1227766	+	**806**	2751	ammonium transporter
hupA	PB7211_929	1129157	1129454	−	**650**	145	non-specific DNA-binding protein HBsu
PB7211_8	PB7211_8	434503	434915	−	**579**	449	ATP synthase subunit C, putative
PB7211_1146	PB7211_1146	381310	382633	−	**554**	655	xanthine/uracil/vitamin C permease family protein
PB7211_669	PB7211_669	529616	530457	+	**507**	265	bacteriorhodopsin
groS	PB7211_1393	400887	401197	−	**496**	340	chaperonin GroS
PB7211_641	PB7211_641	257224	257510	−	**468**	549	conserved hypothetical protein
PB7211_298	PB7211_298	298651	299839	+	**430**	656	trap dicarboxylate transporter - dctp subunit
hflK	PB7211_216	1372461	1373566	−	**426**	631	HflK protein
PB7211_130	PB7211_130	1302141	1303365	+	**423**	395	Receptor family ligand binding domain protein
PB7211_401	PB7211_401	362111	362674	+	**303**	307	acid tolerance regulatory protein actr
acpP	PB7211_217	613491	613746	+	**292**	124	acyl carrier protein
ahcY	PB7211_1045	368453	369739	+	**291**	632	adenosylhomocysteinase
PB7211_767	PB7211_767	1161417	1163849	−	**284**	257	dimethylglycine dehydrogenase
rpoZ	PB7211_373	739908	740423	+	**265**	222	dna-directed rna polymerase omega subunit
gidA	PB7211_100	225271	227142	−	**232**	74	tRNA uridine 5-carboxymethylaminomethyl modification enzyme GidA
yhdW	PB7211_1204	1014159	1015259	−	**215**	1791	ABC transporter
dnaK	PB7211_144	208983	210940	+	**189**	799	chaperone protein DnaK
glcB	PB7211_627	61961	64285	+	**182.5**	86.5	malate synthase
atpD	PB7211_587	338515	340096	+	166	**473**	ATP synthase F1, beta subunit
PB7211_885	PB7211_885	1226899	1228008	+	153	**1799**	conserved hypothetical protein
PB7211_1419	PB7211_1419	904648	906787	+	48	**1253**	V-type H(+)-translocating pyrophosphatase
ftsH	PB7211_115	1396942	1398849	−	25	**2444**	ATP-dependent Zn protease
yjcG	PB7211_576	255410	257224	−	0	**3677**	Na+/solute symporter, Ssf family
groL	PB7211_1188	399202	400863	−	0	**2638**	chaperonin GroL
Tuf	PB7211_560	832167	833357	+	0	**1508**	translation elongation factor Tu
ompA	PB7211_618	1400978	1401475	−	0	**789**	OmpA family protein
aprA	PB7211_563	1114126	1115985	−	0	**606**	adenylylsulfate reductase, alpha subunit
aldA	PB7211_1336	657904	659652	+	0	**577**	acetaldehyde dehydrogenase II
fusA	PB7211_234	845525	847603	+	0	**574**	translation elongation factor G
PB7211_21	PB7211_21	1223550	1224404	+	0	**566**	gxgxg motif-containing protein

The respective number matching this criterion is in boldface and grey-boxed. Genes are ranked according to the reads associated with the respective TSS and then according to the sum of RNA-Seq FW and RV reads (RNA-Seq). The nt positions and gene IDs refer to Supplemental Dataset S2.
