# Supplementary material for: mdRNA-Seq analysis of marine microbial communities from the northern Red Sea
Source: Sci Rep. 2016 Oct 19;6:35470. doi: 10.1038/srep35470 (PMC5069720; doi:10.1038/srep35470)
Supplement: Supplementary Information [file srep35470-s1.pdf]

mdRNA-Seq analysis of marine microbial communities from the northern Red Sea

Shengwei Hou[1], Ulrike Pfreundt[1], Dan Miller[2], Ilana Berman-Frank[2], Wolfgang R Hess[1,*]

[1]*Genetics and Experimental Bioinformatics, Faculty of Biology, University of Freiburg, Schänzlestr. 1, 79104 Freiburg, Germany;*

[2]*Mina and Everard Goodman Faculty of Life Sciences, Bar-Ilan University, Ramat Gan 52900, Israel*

*Corresponding author: wolfgang.hess@biologie.uni-freiburg.de

Additional information

Supporting Methods and Supporting **Figs. S1 – S10** are collected below.

Separately, one Excel workbook is enclosed, with **Supporting Tables S1–S6.**

Supplemental Datasets S1-S6, providing visualizations of the mapping results of transcriptomic data to selected genome or contig sequences and the predicted TSSs, can be downloaded from **https://figshare.com/s/9cc4db8b26bffb94436e**.

Supplemental Dataset S1 Genome wide graphical overview of the mapped reads and coverage for the genome of *Synechococcus* CC9605.

Supplemental Dataset S2 Genome wide graphical overview of the mapped reads and coverage for the genome of SAR11 strain HTCC7211.

Supplemental Dataset S3 Visualization of the details of mapped reads and annotated TSSs on the basis of 17 chromosomes, mitochondrial and chloroplast genomes of *Micromonas* sp. RCC299.

Supplemental Dataset S4 Visualization of the details of mapped reads and annotated TSSs on the basis of 149 environmental contigs belonging to the *Euryarchaeota*.

Supplemental Dataset S5 Alignment of archaeal RNase P RNA sequences including eight euryarchaeal sequences from the Red Sea metatranscriptome.

Supplemental Dataset S6 Visualization of the details of mapped reads and annotated TSSs on the basis of 52 environmental contigs belonging to the *Thaumarchaeota*.

Supplementary Material

Contents

Supplementary Figures 3
Supplementary Methods 12
Supplementary Tables: see separate Excel workbook 23
References 24

Supplementary Figures

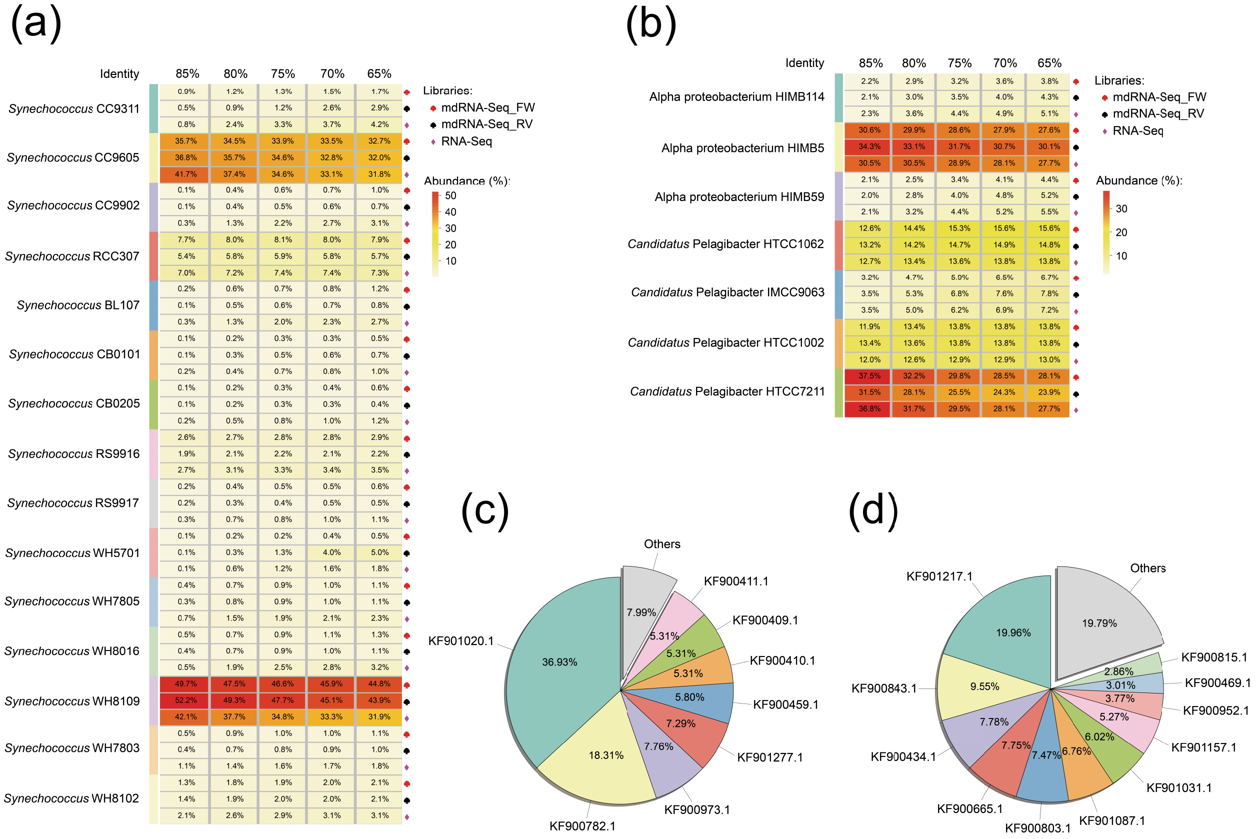

Fig. S1. Distribution of RNA-Seq reads mapping to complete or draft genome sequences. (a) 15 different marine *Synechococcus*[1]; **(b)** 7 different SAR11 strains[2]; **(c)** Contigs of MG-II *Euryarchaeota*[3] or **(d)** marine *Thaumarchaeota*[4].

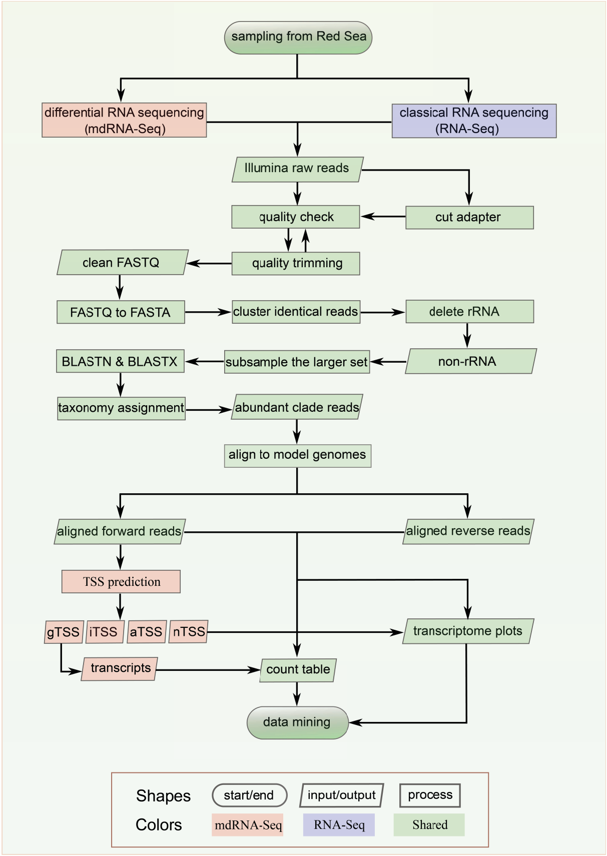

Figure S2. Bioinformatics workflow. Steps specific for mdRNA-Seq, RNA-Seq or shared between both methods are colored in light red, light blue and green, respectively. The sampling and initial processing of RNA samples was described separately[5]. The quality of Illumina fastq raw reads was checked by FastQC v0.10.1[6], adapter sequences were removed using Cutadapt v1.0[7]. Following quality trimming and conversion of FASTQ to FASTA format, computation time was reduced by clustering identical reads in each FASTA file into one representative read. SortMeRNA[8] was used to remove reads matching rRNA and the larger library was subsequently subsampled to get the same number of total reads as from its counterpart. BLASTN and BLASTX v2.2.28+[9] were used to search against NCBI nt and nr databases. BLASTN results were imported into MEGAN v5.2.0[10] for taxonomy assignment. Reads assigned to clades were aligned to possible reference genomes at different identity levels. Segemehl.x v0.1.7-403[11] and TopHat version v2.0.9[12] were used to align the extracted reads to prokaryotic and eukaryotic genomes, respectively. We used the aligned FW reads from mdRNA-Seq to predict and classify potential transcription start sites (TSSs) into gTSS, iTSS, aTSS and nTSS based on genomic context[13,14]. Genome-wide read coverages were visualized in the Artemis genome browser[15], and TSSs were visualized in pdf format. Custom Python, R and Perl scripts were generally used, which are available upon request.

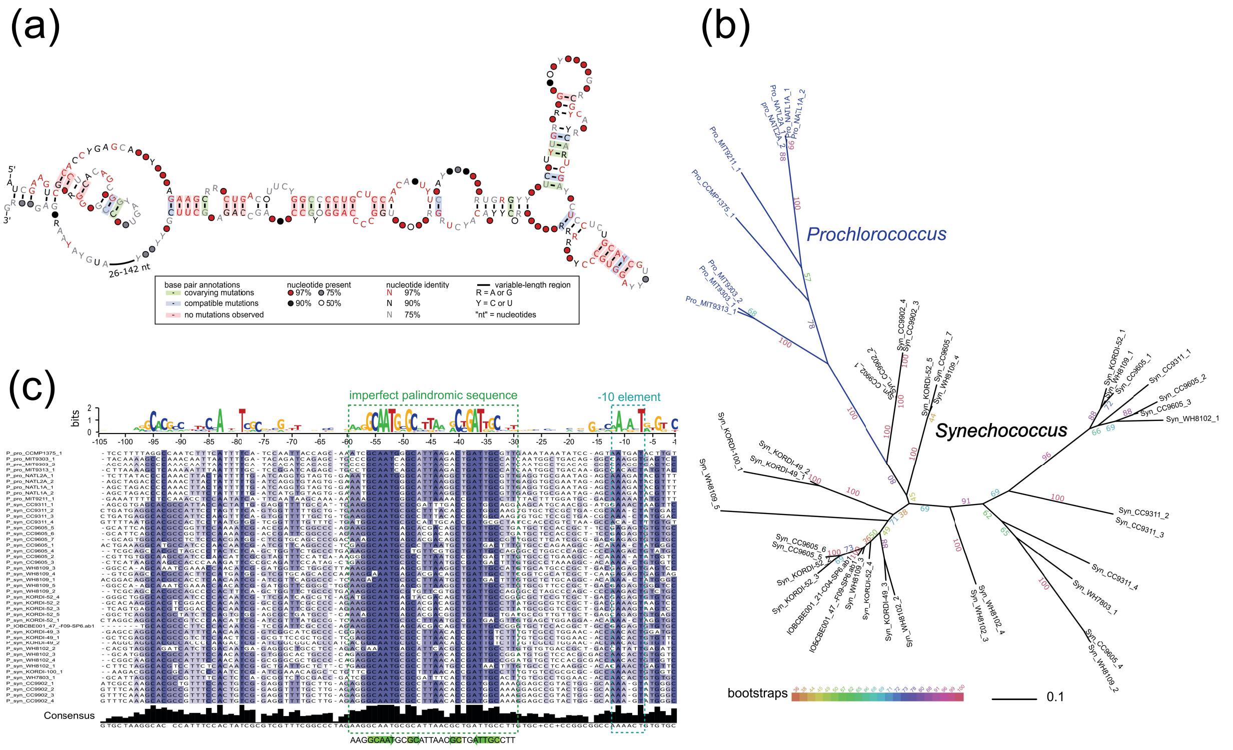

Fig. S3. The non-coding RNA Yfr103. (a) Consensus secondary structure model on the basis of a multiple alignment of 45 sequences from marine *Synechococcus* and *Prochlorococcus*. **(b)** Unrooted phylogenetic tree showing the distribution of Yfr103 among marine picocyanobacteria and the relationship among individual sequences. Bootstrap values are given at branches and color-coded as indicated. **(c)** Multiple sequence alignment of the putative promoter regions upstream of *yfr103* genes. The positions of the -10 element and of a conserved imperfect repeat at positions -30 to -59 are highlighted by the dashed lines. The imperfect palindromic sequence is given at the bottom.

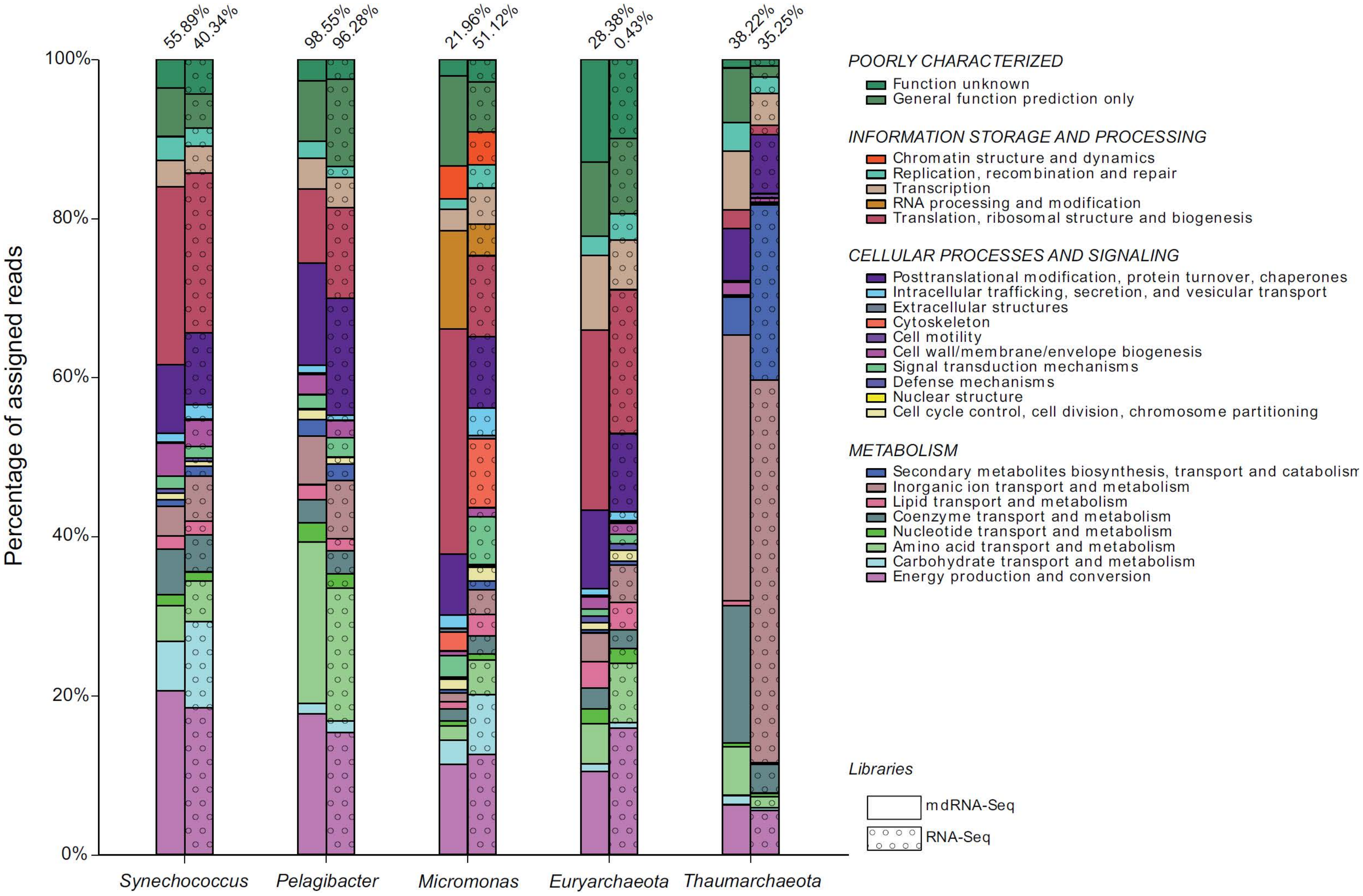

Fig. S4. Comparison of the distribution of 25 major COG categories in mdRNA-Seq and RNA-Seq analysis for *Synechococcus* sp. CC9605, *Candidatus* Pelagibacter sp. HTCC7211, *Micromonas* sp. RCC299 and environmental contigs for MG-II *Euryarchaeota* and *Thaumarchaeota*. The percentage of reads assigned to COG categories from the total number of reads assigned to each organism is given on top of the diagram.

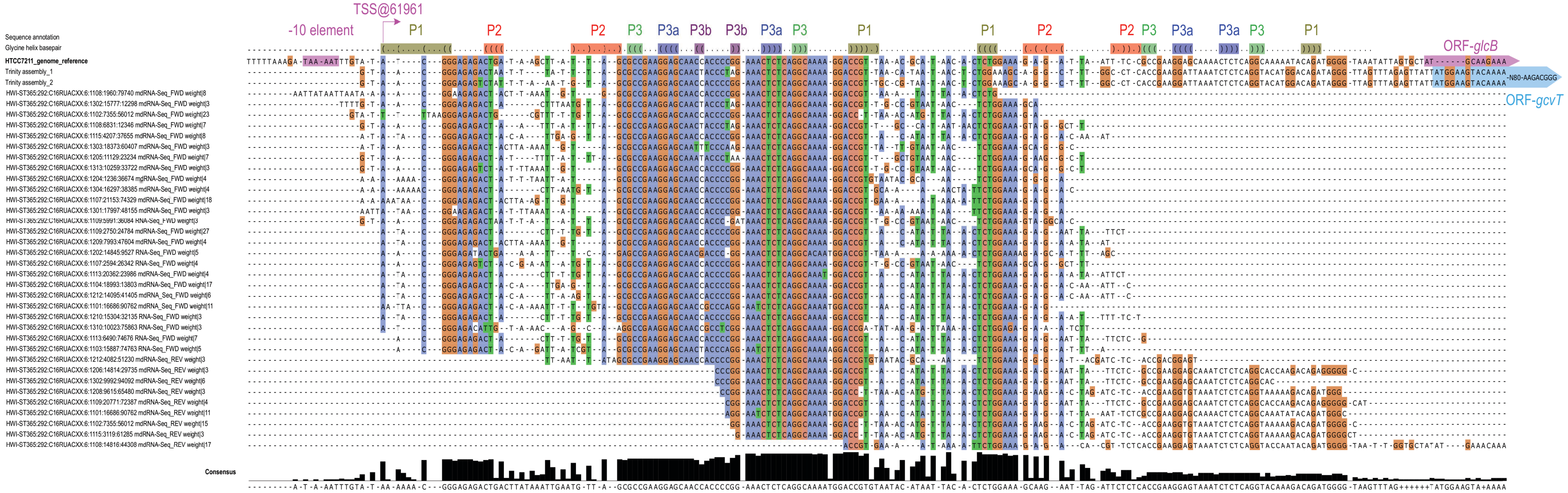

Fig. S5. Details of raw mdRNA-Seq reads covering the SAR11 glycine riboswitch upstream of the *glcB* and *gcvT* genes encoding malate synthase and the glycine cleavage system T protein, respectively. Only the most abundant reads are shown (the weight parameter indicates how often the read was present in the sequenced library). The reference sequence from *Candidatus* Pelagibacter sp. HTCC7211 and two assemblies are drawn above the alignment and the mapped TSS (compare **Dataset S2**) and sequence elements previously defined to constitute the glycine riboswitch[16] are annotated. The start of the *glcB* open reading frame in the reference genome is indicated in pink, the two assemblies obtained from the environmental reads were linked to the start of the *gcvT* gene approximately 80 nt downstream (shadowed in light blue).

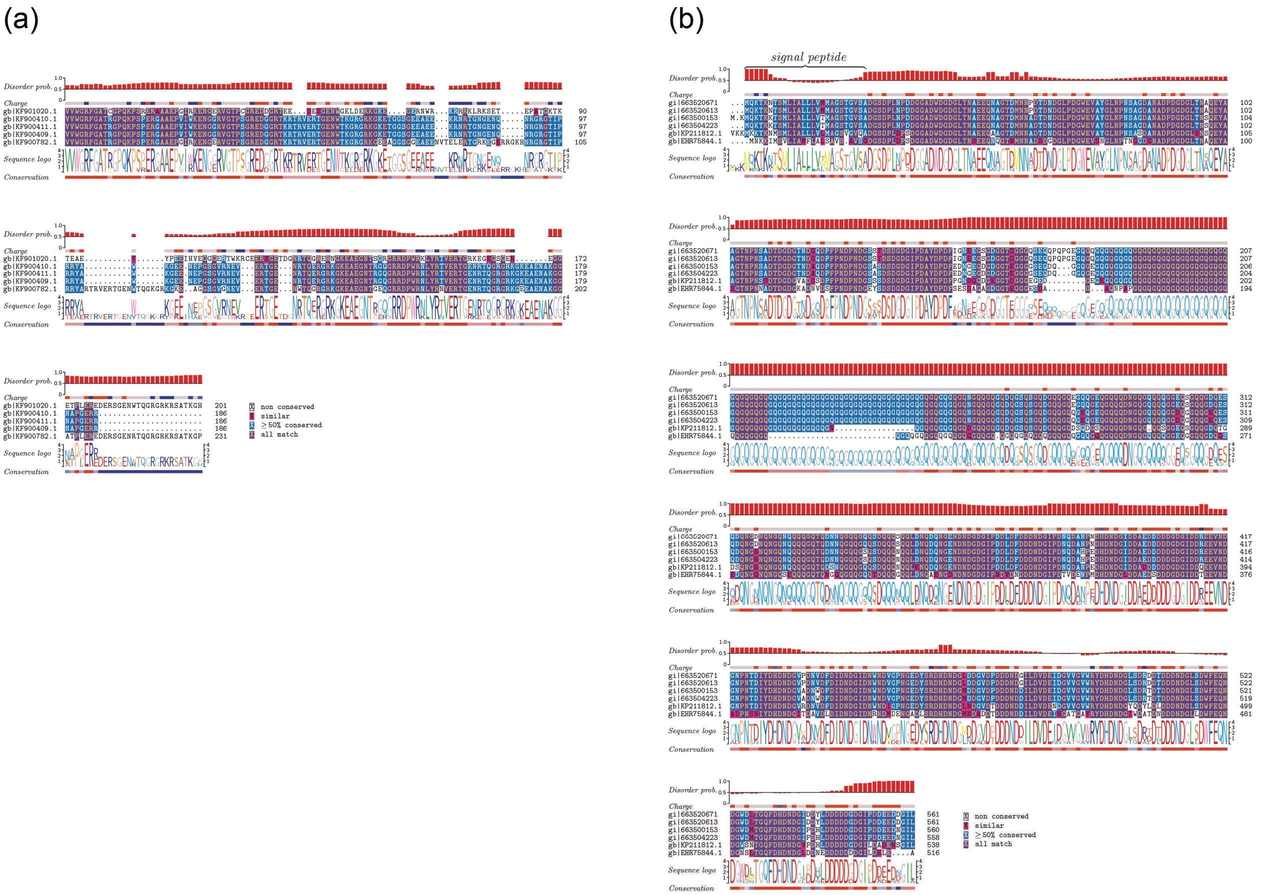

Fig. S6. Two major clusters of intrinsically disordered proteins (IDPs). (a) The first homologous cluster of IDPs. Identifiers on the left show the GenBank accession number of contigs encoding these IDPs. **(b)** The second homologous cluster of IDPs. The first four proteins with GI numbers were detected as highly expressed in this study. Two further homologous proteins were found using BLASTP, from an uncultured *Candidatus* Thalassoarchaea euryarchaeota clone (*gb|KP211812.1*)[3], the other from a metagenome assembly of uncultured MG-II *Euryarchaeota* (*gb|EHR75844.1*)[17]. A transmembrane signal peptide was predicted at their N termini using Phobius[18], followed by a non-cytoplasmic region. *Disorder prob.* shows the disorder probabilities predicted by MetaDisorder[19], the red bars shows deviation of probabilities from the threshold (0.5).

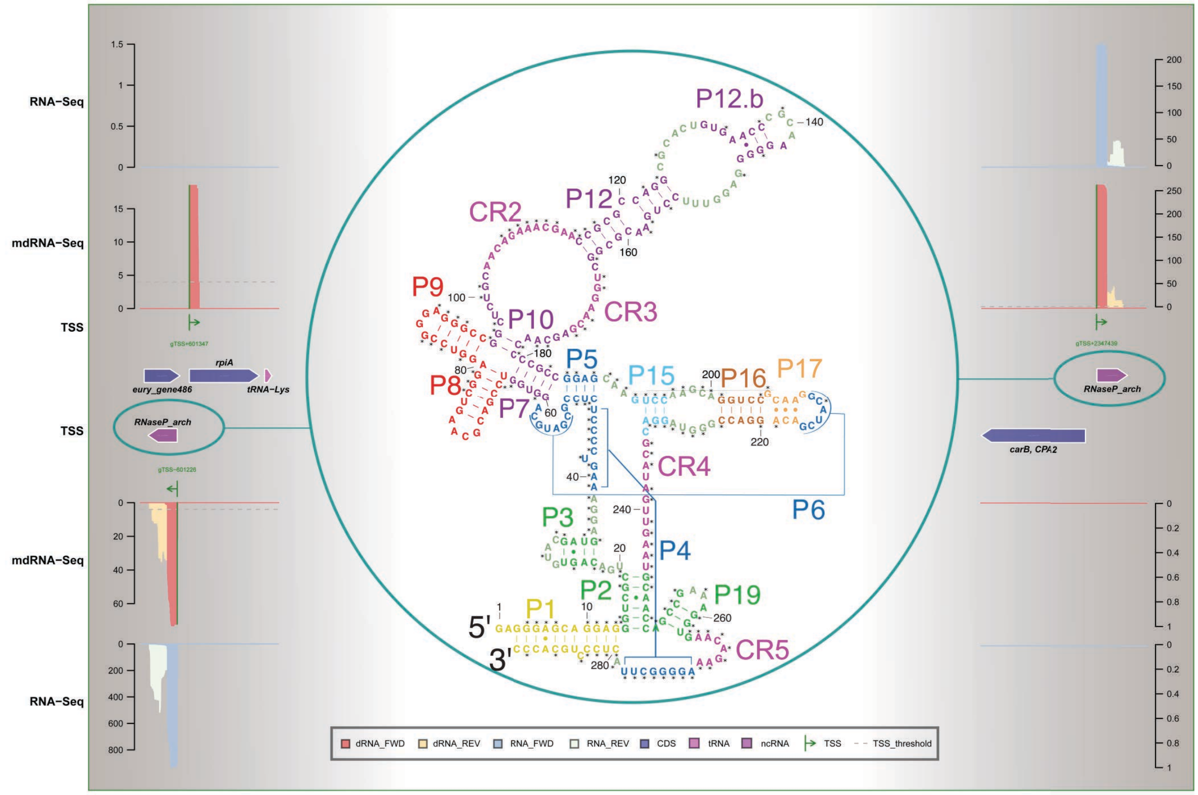

Fig. S7. Identification of RNase P RNA loci in *Euryarchaeota*. Nine of the most highly accumulating euryarchaeal transcripts and most abundant TSSs were found in an antisense orientation to annotated genes. The predicted RNA secondary structure matches the common archaeal type A RNase P[20], whereas the hypothetical gene product has no database matches whatsoever. The RNA secondary structure has been inferred from a sequence-structure comparison of the nine slightly divergent environmental sequences against the archaeal type A RNase P multiple sequence alignment[21]. Within the predicted secondary structure, consensus bases are shown, and positions identical in the nine sequences are labeled using asterisks. The domains P1-P17 and CR1-CR5 represent all essential domains (CR1 is part of P4) of a fully functional RNase P RNA.

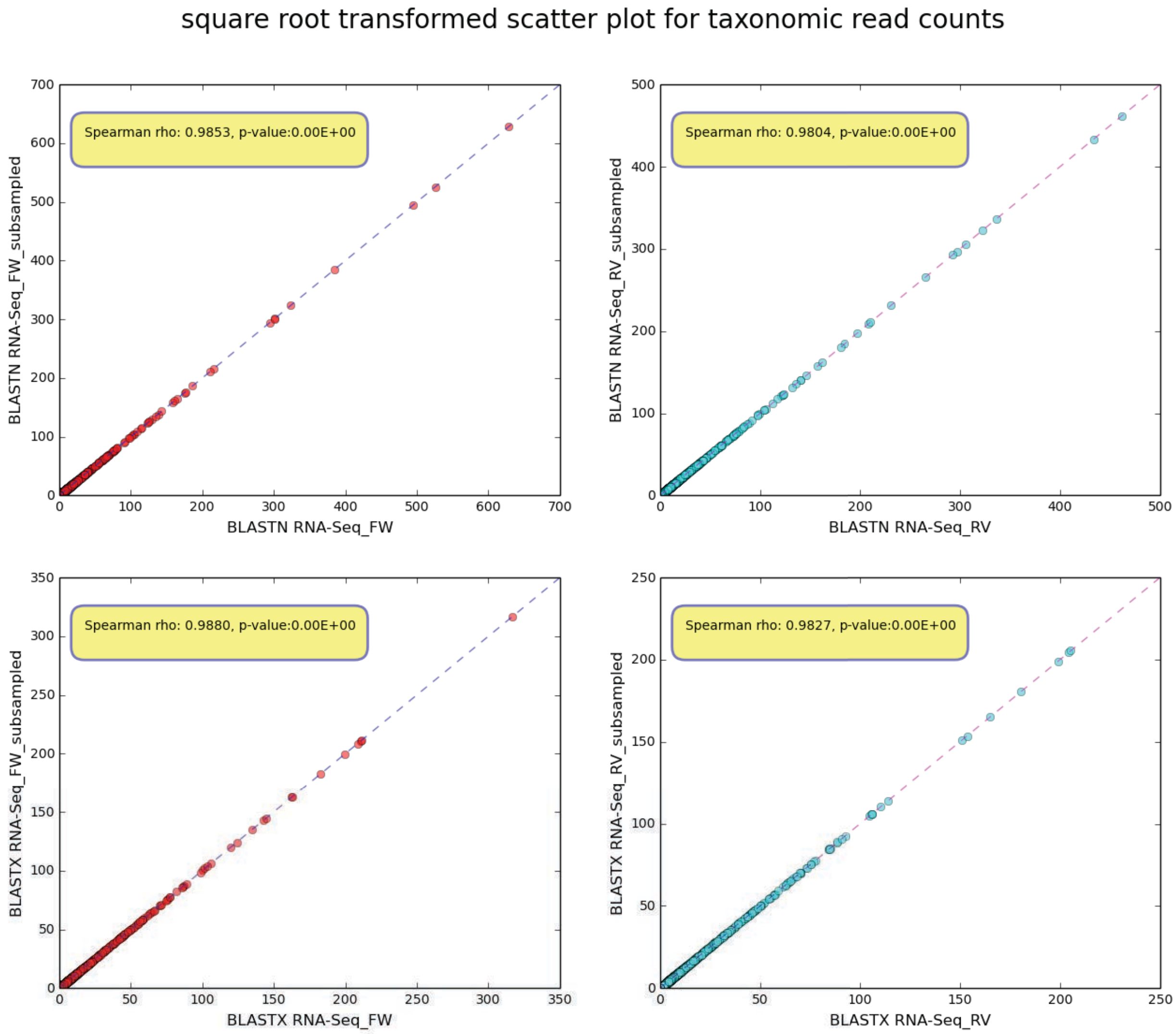

Fig. S8. Correlation of count numbers for taxonomic bins before and after subsampling. Note that all values are square root transformed. The Spearman's rank correlation coefficients as shown in each subplot were calculated based on transformed values. In principle, read counts of technical replicates should correlate well and thus plot along a straight line with slope 1. Therefore, the deviations from this ideal scenario point to systematic differences in transcript detection between the two methods tested here.

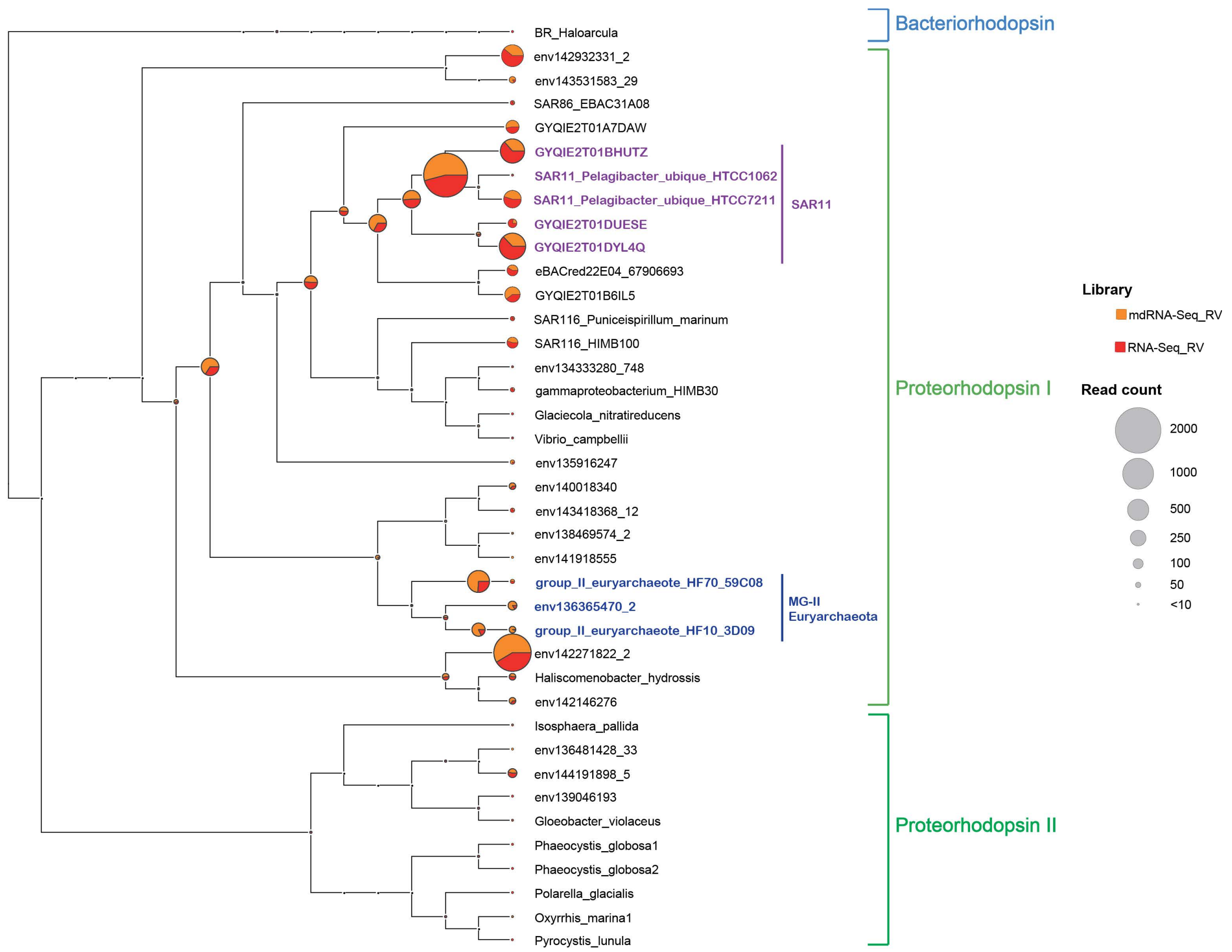

Fig. S9. Phylogenetic distribution of reads assigned to rhodopsin genes. Reference amino acid sequences and the corresponding phylogenetic tree were obtained from the rhodopsin diversity study in the Gulf of Aqaba, Red Sea[22]. Reverse reads from mdRNA-Seq and RNA-Seq libraries were searched against the reference amino acid sequences with an e-value cutoff of 1e-5 using BLASTX. Ambigously assigned reads were passed to their ancestors using MEGAN[22] with LCA parameters: Min Score=30.0, Max Expected=1E-5, Top Percent=2.0, Min Support Percent=0.0(off), Min Support=1, LCA Percent=100 and Min Complexity=0. For clarity, the phylogenetic tree was pruned to keep only nodes with at least one assigned reads in either mdRNA-Seq or RNA-Seq reverse libraries, and was drawn topology only. The sequences were assigned to the three groups *Bacteriorhodopsin*, *Proteorhodopsin I* and *Proteorhodopsin II*, according to references[22,23].

Supplementary Methods

Genome/contig sequences, annotations and database files

Complete or draft genome sequences and annotations were downloaded from ftp://ftp.ncbi.nlm.nih.gov/genomes/ on April 7th, 2014. The *Micromonas* sp. RCC299 genome sequence was downloaded from ftp://ftp.ncbi.nlm.nih.gov/genomes/all/GCF_000090985.2_ASM9098v2 on December 17th, 2014. Marine *Euryarchaeota* and *Thaumarchaeota* environmental contigs[4] were obtained from GenBank using accession numbers KF900301-KF901297 on December 9th, 2014. NCBI nucleotide sequence database (nt) and non-redundant protein sequence database (nr) were downloaded from ftp://ftp.ncbi.nlm.nih.gov/last/db on Sept. 28th, 2014. The MEGAN taxonomy tree file was prepared based on the NCBI taxonomy tree of Dec. 9th, 2014, and corresponding GI to NCBI taxonomy ID conversion files were downloaded from http://ab.inf.uni-tuebingen.de/data/software/megan5/download/welcome.html, which were processed by the MEGAN developer's group based on NCBI taxonomy files of Nov. 21th, 2014. Position-Specific Scoring Matrices profiles of COGs and KOGs were downloaded from the Conserved Domain Database[24]at the NCBI ftp site. The MEROPS[25] database release 10.0 (http://merops.sanger.ac.uk/) was used in the peptidase assignment, and the CAZy[26] database release of July 15th, 2016 was obtained from dbCAN[27] (http://merops.sanger.ac.uk/).

Metatranscriptome analysis - reads cleaning

The major bioinformatics workflow is illustrated in **Figure S2**. The FASTQ format sequence files were first checked by FastQC v0.10.1[6] with default parameters, then Cutadapt v1.0[7] was applied to remove adapter sequences ("-b" option), with 10 nt minimum overlap (-"O" option), 0.2 minimum allowed error rate ("-e" option). This adapter removal step was repeated 5 times ("-n" option). Low quality sequences in the 3' end of each read were trimmed by fastq_quality_trimmer, with ASCII codes of Phred score starting from 33 ("-Q" option), a minimum quality threshold of 20 ("-t" option), and a minimum length threshold of 20 ("-l" option). After quality trimming, the FASTQ files were converted into FASTA format by applying fastq_to_fasta, with the same ASCII codes offset from 33 ("-Q" option), and keeping reads with "N"s ("-n" option). Both fastq_quality_trimmer and fastq_to_fasta are parts of FASTX-Toolkit, v0.0.13 (available at http://hannonlab.cshl.edu/fastx_toolkit/). To reduce computation time, identical reads in each FASTA file were clustered into one representative read, with a weight field in the header of each read indicating the total reads number in each unique cluster. To remove rRNAs, the dababases supplied by SortMeRNA v1.9[8] were merged into a single FASTA file, which was used as a database file ("-db" option) to classify the reads into rRNAs and non-rRNAs with default parameters.

Metatranscriptome analysis – subsampling and validation

To assess coverage of various members of the microbial community, we first sequenced total RNA without further depletion or enrichment of certain RNA classes[24], using Illumina technology. After trimming adaptors and removing low quality and ribosomal RNA reads (**Figure S2**), mdRNA-Seq and RNA-Seq yielded more than 6

million read pairs each (**Table S1**). To compare the performance of the two different techniques quantitatively, we adjusted the library sizes by random subsampling to the smaller set of the libraries, discarding 7.41% of the forward (FW) reads and 9.82% of the reads in reverse orientation (RV) from the RNA-Seq library.

To test that the subsampling process was unbiased, we imported the BLASTN and BLASTX results of the subsampled reads and the reads before subsampling into MEGAN, then the read count assigned to each taxonomic node was collected into a taxonomic count table. The count numbers were further normalized against the respective library size, then plotted in square root scale and the Spearman's Rank-Order correlation coefficients were calculated (**Figure S8**).

Metatranscriptome analysis – taxonomic assignment

BLASTN and BLASTX v2.2.28+[9] were used to search the unique clustered reads against NCBI nt and nr databases. The BLASTN results were imported into MEGAN v5.2.0[29] for taxonomic assignment with the following lowest common ancestor parameters: Min Score=50.0, Max Expected=1E-5, Top Percent=1.0, Min Support Percent= 0.0(off), Min Support=2, LCA Percent=100.0, Min Complexity=0.25. Within MEGAN, reads belonging to "*Synechococcus*" and its descendants, as well as reads assigned to ancestor nodes ("*Chroococales*", "*Oscillatoriophycideae*", "*Cyanobacteria*", "*Bacteria*", "*Cellular organisms*") were extracted together as read set "Cellular2Synechococcus". Reads belonging to "*SAR11 cluster*" and its descendants, as well as reads assigned to ancestor nodes ("*unclassified Alphaproteobacteria*", "*Alphaproteobacteria*", "*Proteobacteria*", "*Bacteria*") were extracted together as read set

“Bac2SAR11cluster”, reads assigned to “*Archaea*” and all its descendants were extracted together as “ArchaeaSum”, and reads assigned to “*Mamiellales*” and its descendants, as well as to ancestor nodes (“*Mamiellophyceae*”, “*Prasinophytes*”, “*Chlorophyta*”, “*Viridiplantae*”, “*Eukaryota*”, “*Cellular organisms*”) were extracted as read set “Cellular2Mamiellales”.

Model reference genome selection

To find suitable reference genomes for each relatively abundant read set, all complete and draft genomes for the respective clades were downloaded from the NCBI ftp site, then the corresponding set of reads were aligned to these genomes at identity cut-offs of 85%, 80%, 75%, 70% and 65%. Segemehl.x v0.1.7-403[11] was used to align prokaryotic reads to their reference genomes with specified identity (“-A“ option), minimum reads length of 20 (“-m“ option), and other default parameters. Those complete genome sequences recruiting a maximum of reads were selected as references for the subsequent metatranscriptome analysis. Because most archaeal reads were recruited by contigs of uncultured MG-II/III *Euryarchaeota* from a metagenomic library[4], all the “ArchaeaSum” reads were aligned to these contigs at 75% identity using Segemehl.x. Finally, 201 contigs were selected that each recruited at least 100 reads totally in mdRNA-Seq and RNA-Seq. From these, 149 contigs were ascribed to MG-II/III *Euryarchaeota* (total of 5.4 Mb), and 52 contigs were classified as *Thaumarchaeota* (total of 1.9 Mb). These two sets of contigs were further merged into two concatenated reference genome scaffolds, with 100 “N”s separating each two neighboring contigs.

We chose the chromosome, mitochondrial and chloroplast sequences of *Micromonas* sp. RCC299[30] to represent the Mamelliaceae. TopHat version v2.0.9[12] was used to align the extracted eukaryotic reads to *Micromonas* chromosomes with following parameters: --b2-D=20, --b2-R=3, --b2-N=1, --b2-L=20, --b2-i=S,1,0.5, --read-mismatches=10, --read-gap-length=10, --read-edit-dist=15, --read-realign-edit-dist=0, --min-anchor-length=5, --splice-mismatches=1, --max-multihits=20, --microexon-search, --segment-mismatches=2, --segment-length=20, --no-convert-bam. Based on the analysis of the MD field in the output SAM files, these parameters will produce a minimum identity of 77% for both orientations of dRNA-Seq reads, and 74% for RNA-Seq FW library, 75% for RNA-Seq RV library, which is comparable to the 75% identity we used in Segemehl.x.

Prediction of transcriptional start sites (TSSs)

TSS prediction was done for all reference sequences. mdRNA-Seq reads from environmental populations map to representative genomes more fuzzy than reads from cultured microorganisms. Thus, a few additional parameters were defined to optimize TSS prediction for the metatranscriptomic analysis (**Figure S10).** We defined **d**RNA-Seq **F**orward **R**ead **S**tart **S**ites (hereafter dFRSS) as genome positions where transcripts start. Due to the sequence diversity of the metatranscriptomic data, noisy dFRSS can be expected around the TSS of any given transcript. Thus, all dFRSS in a clustering range *R* around a local maximum dFRSS were clustered into raw TSS (**Figure S10,** *Clustering*), using a list of all dFRSS sorted after descending expression strength, with *TSS-count* as the number of reads clustered to this position. A similar

approach was used before also in culture-centered studies to account for the natural fuzziness of some TSSs[13,14]. These raw TSS were classified into preliminary gTSS, iTSS, aTSS and nTSS. Specifically, a TSS was classified as a gTSS if it was localized ≤200 nt upstream of a gene (≤600 nt for *Micromonas* sp. RCC299 chromosomes), or if it was located >200 nt upstream region of a gene but the mean read coverage of mdRNA-Seq and RNA-Seq starting at this TSS overlapped with this gene; an iTSS was defined when a TSS was located within a gene, an aTSS was defined when a TSS was located antisense to the coding region of a gene or within 50 nt of its 5' or 3'UTRs. When a TSS didn't match any of the above scenarios, an nTSS was defined. In addition, a TSS driving transcription of a previously annotated gene for a non-coding RNA (e.g., rRNA, tRNA, rnpB) was also defined as nTSS. These TSS groups were mutually exclusive, with hierarchical assignments (gTSS > iTSS > aTSS > nTSS).

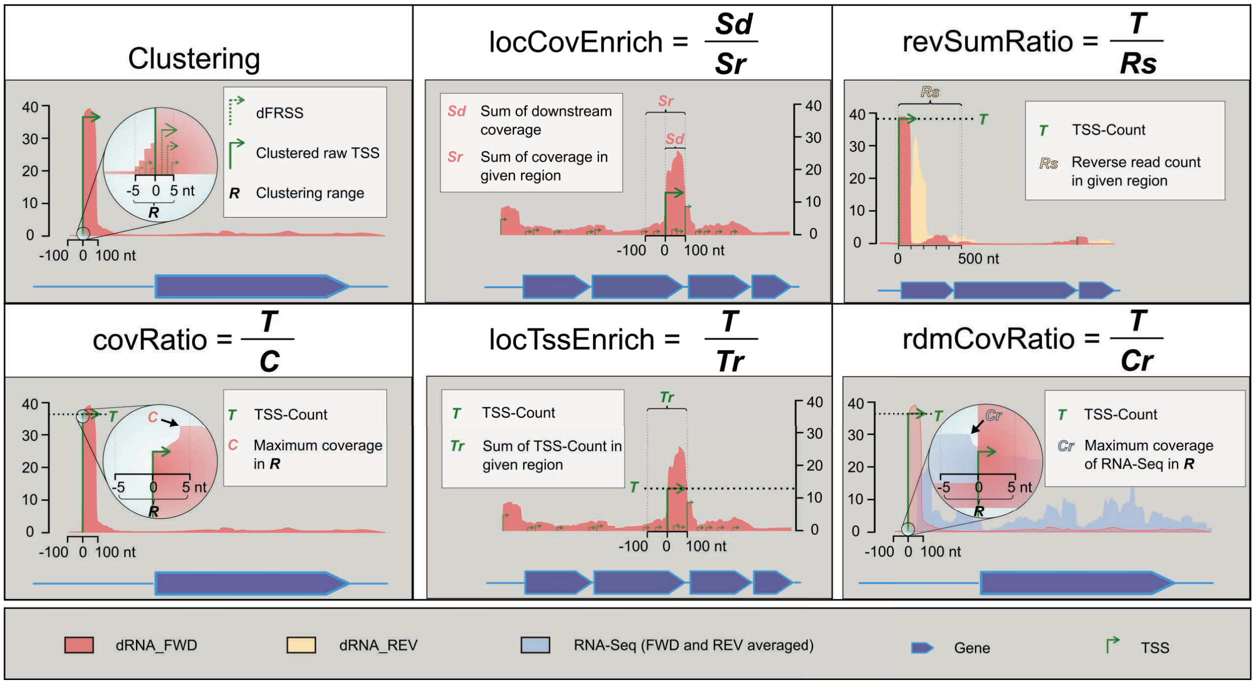

Fig. S10. Schematic explanation of TSS prediction parameters optimized for mdRNA-Seq metatranscriptomic data. First, noisy **dFRSS** were clustered 5 nt upstream and 5 nt downstream of the local maximum to create a raw TSS. The ***covRatio*** is defined as the ratio between the read count at each

raw TSS and the maximum background coverage in the clustering window[31]. The local coverage enrichment score, ***locCovEnrich***, was calculated as the ratio between the coverage 100 nt downstream of a raw TSS (same as read length) and the coverage in a 200 nt window around it. The local TSS enrichment score (modified after[32]), ***locTssEnrich***, was calculated as the ratio between the *TSS-count* at a raw TSS and the sum of all *TSS-counts* in a 200 nt window. The ***revSumRatio*** was defined as the ratio between the *TSS-count* at a raw TSS and the sum of the mapped RV reads from the TSS to 500 nt downstream (same as maximum fragment size of library preparation). The ***rdmCovRatio*** was defined as the ratio between the *TSS-count* at a raw TSS and the maximum RNA-Seq coverage in the clustering window. RNA-Seq coverage per position was calculated as the average between the FW and RV read coverage at that position.

Besides *TSS-count*, another three basic parameters were used to screen the preliminary TSSs for possible false-positives. First, the *TSS-count* was divided by the maximum coverage value (of mdRNA-Seq FW reads) in a 10 nt window around the TSS (**Figure S10,** *covRatio*), to discard TSS too close to the surrounding noise[31]. Second, a local coverage enrichment score (**Figure S10,** *locCovEnrich*) was calculated to discard weak secondary TSSs downstream of a stronger TSS. Third, a local TSS enrichment score (**Figure S10,** *locTssEnrich*), previously defined in the TSSer program[32], was applied with modifications to make it more sensitive to surrounding raw TSSs.

An additional parameter was introduced to account for a specific situation arising in metatranscriptomic datasets. Due to conserved sequences, a region might recruit FW reads but none or little RV reads. Therefore, the ratio of *TSS-count* to total RV read number ensured removal of TSSs without sufficient RV read support (**Figure S10,** *revSumRatio*). Further and only applied to weaker TSSs below 100 *TSS-counts*, we introduced a parameter to discard false-positive TSSs likely arising from fragmentation bias due to RNA secondary structures instead of true transcript starts. An indication for this is high coverage in the RNA-Seq library, but a weak *TSS-count* (**Figure S10,** *rdmCovRatio*). Values for these parameters were determined for each reference

genome by iterative application of parameter-sets and manual inspection of discarded TSS.

Genome-wide read coverage was visualized in the Artemis genome browser[15], TSS distributions and coverage data were visualized and inspected with R. Finally, TSS prediction parameters were as follows. *Synechococcus* sp. CC9605: *covRatio*>0.5, *locCovEnrich*>0.65, *locTssEnrich*>0.4, *revSumRatio*<=10, *rdmCovRatio*>0.85. *Candidatus* Pelagibacter sp. HTCC7211: *covRatio*>0.5, *locCovEnrich*>0.5, *locTssEnrich*>0.3, *revSumRatio*<=30, rdm*CovRatio*>0.85. *Micromonas* sp. RCC299: *covRatio*>0.5, *locCovEnrich*>0.5, *locTssEnrich*>0.3, rdm*CovRatio*>0.5. *revSumRatio* was not applied to *Micromonas*, because the distance between FW and RV reads could be more than 500 nt due to the presence of introns. *Euryarchaeota* and *Thaumarchaeota*: same parameters as for *Candidatus* Pelagibacter sp. HTCC7211, except for *revSumRatio*, which was not applied because of homologous genes in different contigs. We chose 13 as minimum *TSS-count* for *Synechococcus* sp. CC9605 and a cutoff of 4 for the other genomes to compensate for the lower coverage. After TSS prediction, transcripts were defined as the sequence from a gTSS to the end of the first following gene in sense orientation and the reads mapping to it were counted to generate a count table.

Systematic comparison of mRNA detection by mdRNA-Seq or RNA-Seq for Synechococcus

A count table was generated for every gene in the *Synechococcus* sp. CC9605 genome. For mdRNA-Seq, the *TSS-count* at each gTSS was used. For both mdRNA-

Seq and RNA-Seq, all reads mapping to the transcripts were counted. All genes with 2 reads minimum in both libraries were counted as “detected with both methods”, those with 1 read or none in one library and ≥2 reads in the other were counted as “detected with only one method”. Excluding singleton read counts is the most cautious cut-off possible. We did not use any higher cut-offs for this analysis due to the generally low coverage for single-species transcriptomes within the metatranscriptome. For all genes detected with both methods, the $\log_{10}$ read counts from both libraries were plotted against each other in a scatter plot to visualize their correlation using the ggplot2 package in R[33]. Using the *lm* function, a linear model with a confidence level of 0.95 was fitted to the data (**Figure S8**).

ncRNA analysis: alignment, promoter motif, secondary structure

MAFFT (v7.221) E-INS-i[34] or T-Coffee (v11.00.8cbe486)[35] were used for multiple sequence alignments of selected ncRNA homologs with default parameters. Secondary structures were predicted by the RNAalifold[36] online server with default parameters , then plotted using R2R[37]**.** The alignments with secondary structure information were subjected to R2R, v1.0.4[37], to generate consensus plots. To build a phylogenetic tree, nucleotide positions were removed, if more than 40% of the alignment at this position were gaps and not basepairing with another position. RAxML v8.1.20[38] was used for maximum likelihood unrooted tree construction, with the default GTR S16 state dinucleotide substitution model for paired nucleotides (“-A”=S16), and default GTR nucleotide substitution model for unpaired nucleotides, with GAMMA model of rate heterogeneity (“-m”=GTRGAMMA). A fixed random number seed 12345 (“-x” option) was provided to force RAxML generating the same starting tree every time (“-p” option),

1000 rapid boostrap searches (every 5th bootstrap tree was used as a starting point to search for maximum likelihood trees as documented in RAxML manual) were performed. The resulting unrooted tree was visualized in FigTree v1.4.2 (available at http://tree.bio.ed.ac.uk/software/figtree/).

COG/peptidases/carbohydrate-active enzymes assignments, definition of intrinsically disordered proteins and characterization of the glycine riboswitch RPSBLAST from the NCBI BLAST toolkit v2.2.31+ was used to analyse protein sequences against COGs/KOGs PSSM profiles of the Conserved Domain Database[24], and the hit with the best e-value was taken. mdRNA-Seq and RNA-Seq read counts assigned to transcripts were assigned to COG/KOG clusters. By querying the functional categories within each COG/KOG cluster, read counts were split to 25 functional categories. Counts assigned to more than one category were distributed equally. Finally, the generated count tables of 25 COG functional categories were merged and visualized using matplotlib[39].

To get categorical annotation of peptidases and carbohydrate-active enzymes, BLASTP was used to assign proteins of selected references against the MEROPS[25] and CAZy[26] databases with an e-value cutoff of 1e-10, and the annotations of the best hits were taken. Extracelluar peptidases were identified when a signal peptide was predicted using the online server of SignalP v4.1[40] with default parameters.

Intrinsically disordered proteins (IDPs) were predicted using MetaDisorder[19], which collects primary disorder predictions from 15 other predictors, then weight each

prediction based on method accuracy to produce a final prediction. Homologs of IDPs were aligned using T-Coffee with default parameters, and plotted using TexShade[41].

FW reads mapped to the glycine riboswitch were extracted from mdRNA-Seq and RNA-Seq read files together with their corresponding RV reads. The paired FW and RV reads were assembled using Trinity v2.0.6[42] with --seqType=fa, --SS_lib_type=FR, and other default parameters.

Supplementary Tables: see separate Excel workbook

Supplementary Table S1 Sequencing facts, rRNA depletion, and sequence read redundancy. Given are the total and unique numbers of reads in each category and the mean number of identical reads in a unique cluster (mean weight). Note the higher redundancy of sequence reads (100% identity) in the mdRNA-Seq FW library due to selective sequencing of nascent 5' ends.

Supplementary Table S2 Details and classification of all predicted TSSs according to the mapping of mdRNA-Seq reads to the *Synechococcus* CC9605 genome and RNA-Seq read coverage. Given is the gene name, locus tag ID, the TSS ID, start and end of the transcript, genome strand, the read counts defining the TSS, and read counts for all four libraries associated with the TSS or transcriptional unit. Moreover, if known, the gene product is given followed by the sum of reads for all four libraries and for the RNA-Seq libraries and by a link to the respective entry in NCBI if available.

Supplementary Table S3 Protein-coding genes that were re-annotated, corrected or newly defined based on the metatranscriptome mapping results to the *Synechococcus* sp. CC9605 genome. Newly defined genes are Syn_n004 to Syn_n083.

Supplementary Table S4 Mapped reads and annotated TSSs for *Micromonas* sp. RCC299 chromosomes and organellar genomes. Columns are labelled as in **Table S1**.

Supplementary Table S5 Details of mapped reads and annotated TSSs for MG-II/III *Euryarcheota* based on 149 contigs.

Supplementary Table S6 Details of mapped reads and annotated TSSs for marine *Thaumarcheota* based on 52 selected contigs.

References

1. Scanlan, D. J. *et al.* Ecological genomics of marine picocyanobacteria. *Microbiol. Mol. Biol. Rev.* **73,** 249–299 (2009).
2. Grote, J. *et al.* Streamlining and core genome conservation among highly divergent members of the SAR11 clade. *mBio* **3,** e00252–12 (2012).
3. Martin-Cuadrado, A.-B. *et al.* A new class of marine Euryarchaeota group II from the mediterranean deep chlorophyll maximum. *ISME J.* **9,** 1619–1634 (2015).
4. Deschamps, P., Zivanovic, Y., Moreira, D., Rodriguez-Valera, F. & López-García, P. Pangenome evidence for extensive interdomain horizontal transfer affecting lineage core and shell genes in uncultured planktonic Thaumarchaeota and Euryarchaeota. *Genome Biol. Evol.* **6,** 1549–1563 (2014).
5. Pfreundt, U. *et al.* Depth dependent metatranscriptomes of the marine pico-/nanoplanktonic communities in the Gulf of Aqaba/Eilat during seasonal deep mixing. *Mar. Genomics* **18,** 93–95 (2014).
6. Andrews, S. FastQC A quality control tool for high throughput sequence data. (2010). Available at: http://www.bioinformatics.babraham.ac.uk/projects/fastqc/. (Accessed: 14th July 2014)
7. Martin, M. Cutadapt removes adapter sequences from high-throughput sequencing reads. *EMBnet.journal* **17,** 10 (2011).
8. Kopylova, E., Noe, L. & Touzet, H. SortMeRNA: fast and accurate filtering of ribosomal RNAs in metatranscriptomic data. *Bioinformatics* **28,** 3211–3217 (2012).
9. Altschul, S. F., Gish, W., Miller, W., Myers, E. W. & Lipman, D. J. Basic local alignment search tool. *J. Mol. Biol.* **215,** 403–410 (1990).
10. Huson, D. H., Mitra, S., Ruscheweyh, H.-J., Weber, N. & Schuster, S. C. Integrative analysis of environmental sequences using MEGAN4. *Genome Res.* **21,** 1552–1560 (2011).

11. Hoffmann, S. *et al.* Fast mapping of short sequences with mismatches, insertions and deletions using index structures. *PLoS Comput Biol* **5,** e1000502 (2009).
12. Trapnell, C., Pachter, L. & Salzberg, S. L. TopHat: discovering splice junctions with RNA-Seq. *Bioinformatics* **25,** 1105–1111 (2009).
13. Mitschke, J., Vioque, A., Haas, F., Hess, W. R. & Muro-Pastor, A. M. Dynamics of transcriptional start site selection during nitrogen stress-induced cell differentiation in Anabaena sp. PCC7120. *Proc. Natl. Acad. Sci. U. S. A.* **108,** 20130–20135 (2011).
14. Mitschke, J. *et al.* An experimentally anchored map of transcriptional start sites in the model cyanobacterium Synechocystis sp. PCC6803. *Proc. Natl. Acad. Sci. U. S. A.* **108,** 2124–2129 (2011).
15. Rutherford, K. *et al.* Artemis: sequence visualization and annotation. *Bioinformatics* **16,** 944–945 (2000).
16. Tripp, H. J. *et al.* Unique glycine-activated riboswitch linked to glycine-serine auxotrophy in SAR11. *Environ. Microbiol.* **11,** 230–238 (2009).
17. Iverson, V. *et al.* Untangling Genomes from Metagenomes: Revealing an Uncultured Class of Marine Euryarchaeota. *Science* **335,** 587–590 (2012).
18. Käll, L., Krogh, A. & Sonnhammer, E. L. L. A combined transmembrane topology and signal peptide prediction method. *J. Mol. Biol.* **338,** 1027–1036 (2004).
19. Kozlowski, L. P. & Bujnicki, J. M. MetaDisorder: a meta-server for the prediction of intrinsic disorder in proteins. *BMC Bioinformatics* **13,** 111 (2012).
20. Harris, J. K., Haas, E. S., Williams, D., Frank, D. N. & Brown, J. W. New insight into RNase P RNA structure from comparative analysis of the archaeal RNA. *RNA N. Y. N* **7,** 220–232 (2001).
21. Brown, J. W. The Ribonuclease P Database. *Nucleic Acids Res.* **27,** 314–314 (1999).
22. Philosof, A. & Béjà, O. Bacterial, archaeal and viral-like rhodopsins from the Red Sea. *Environ. Microbiol. Rep.* **5,** 475–482 (2013).

23. Yutin, N. & Koonin, E. V. Proteorhodopsin genes in giant viruses. *Biol. Direct* **7,** 34 (2012).

24. Marchler-Bauer, A. *et al.* CDD: NCBI's conserved domain database. *Nucleic Acids Res.* **43,** D222–226 (2015).

25. Rawlings, N. D., Barrett, A. J. & Bateman, A. MEROPS: the database of proteolytic enzymes, their substrates and inhibitors. *Nucleic Acids Res.* **40,** D343–D350 (2012).

26. Lombard, V., Ramulu, H. G., Drula, E., Coutinho, P. M. & Henrissat, B. The carbohydrate-active enzymes database (CAZy) in 2013. *Nucleic Acids Res.* **42,** D490–D495 (2014).

27. Yin, Y. *et al.* dbCAN: a web resource for automated carbohydrate-active enzyme annotation. *Nucleic Acids Res.* **40,** W445–W451 (2012).

28. Sharma, C. M. *et al.* The primary transcriptome of the major human pathogen Helicobacter pylori. *Nature* **464,** 250–255 (2010).

29. Huson, D. H., Auch, A. F., Qi, J. & Schuster, S. C. MEGAN analysis of metagenomic data. *Genome Res.* **17,** 377–386 (2007).

30. Worden, A. Z. *et al.* Green evolution and dynamic adaptations revealed by genomes of the marine picoeukaryotes Micromonas. *Science* **324,** 268–272 (2009).

31. Pfreundt, U., Kopf, M., Belkin, N., Berman-Frank, I. & Hess, W. R. The primary transcriptome of the marine diazotroph Trichodesmium erythraeum IMS101. *Sci. Rep.* **4,** 6187 (2014).

32. Jorjani, H. & Zavolan, M. TSSer: an automated method to identify transcription start sites in prokaryotic genomes from differential RNA sequencing data. *Bioinforma. Oxf. Engl.* **30,** 971–974 (2014).

33. Wickham, H. *ggplot2*. (Springer New York, 2009).

34. Katoh, K. & Standley, D. M. MAFFT Multiple Sequence Alignment Software Version 7: Improvements in Performance and Usability. *Mol. Biol. Evol.* **30,** 772–780 (2013).

35. Notredame, C., Higgins, D. G. & Heringa, J. T-coffee: a novel method for fast and accurate multiple sequence alignment1. *J. Mol. Biol.* **302,** 205–217 (2000).

36. Bernhart, S. H., Hofacker, I. L., Will, S., Gruber, A. R. & Stadler, P. F. RNAalifold: improved consensus structure prediction for RNA alignments. *BMC Bioinformatics* **9,** 474 (2008).

37. Weinberg, Z. & Breaker, R. R. R2R - software to speed the depiction of aesthetic consensus RNA secondary structures. *BMC Bioinformatics* **12,** 3 (2011).

38. Stamatakis, A. RAxML version 8: a tool for phylogenetic analysis and post-analysis of large phylogenies. *Bioinformatics* **30,** 1312–1313 (2014).

39. Hunter, J. D. Matplotlib: A 2D Graphics Environment. *Comput. Sci. Eng.* **9,** 90–95 (2007).

40. Petersen, T. N., Brunak, S., von Heijne, G. & Nielsen, H. SignalP 4.0: discriminating signal peptides from transmembrane regions. *Nat. Methods* **8,** 785–786 (2011).

41. Beitz, E. TeXshade: shading and labeling of multiple sequence alignments using LaTeX2e. *Bioinformatics* **16,** 135–139 (2000).

42. Grabherr, M. G. *et al.* Full-length transcriptome assembly from RNA-Seq data without a reference genome. *Nat. Biotechnol.* **29,** 644–652 (2011).
